# Combination of resveratrol and 5‐azacytydine improves osteogenesis of metabolic syndrome mesenchymal stem cells

**DOI:** 10.1111/jcmm.13731

**Published:** 2018-07-12

**Authors:** Krzysztof Marycz, Katarzyna Kornicka, Jennifer M. Irwin‐Houston, Christine Weiss

**Affiliations:** ^1^ Department of Experimental Biology Wroclaw University of Environmental and Life Sciences Wroclaw Poland; ^2^ Wroclaw Research Centre EIT+ Wrocław Poland; ^3^ PferdePraxis Dr. Med. Vet. Daniel Weiss Freienbach Switzerland

**Keywords:** adipose stem cells, ageing, autophagy, metabolic syndrome, mitochondria, osteogenic differentiation

## Abstract

Endocrine disorders have become more and more frequently diagnosed in humans and animals. In horses, equine metabolic syndrome (EMS) is characterized by insulin resistance, hyperleptinemia, hyperinsulinemia, inflammation and usually by pathological obesity. Due to an increased inflammatory response in the adipose tissue, cytophysiological properties of adipose derived stem cells (ASC) have been impaired, which strongly limits their therapeutic potential. Excessive accumulation of reactive oxygen species, mitochondria deterioration and accelerated ageing of those cells affect their multipotency and restrict the effectiveness of the differentiation process. In the present study, we have treated ASC isolated from EMS individuals with a combination of 5‐azacytydine (AZA) and resveratrol (RES) in order to reverse their aged phenotype and enhance osteogenic differentiation. Using SEM and confocal microscope, cell morphology, matrix mineralization and mitochondrial dynamics were assessed. Furthermore, we investigated the expression of osteogenic‐related genes with RT‐PCR. We also investigated the role of autophagy during differentiation and silenced PARKIN expression with siRNA. Obtained results indicated that AZA/RES significantly enhanced early osteogenesis of ASC derived from EMS animals. Increased matrix mineralization, RUNX‐2, collagen type I and osteopontin levels were noted. Furthermore, we proved that AZA/RES exerts its beneficial effects by modulating autophagy and mitochondrial dynamics through PARKIN and RUNX‐2 activity.

## 1 INTRODUCTION

Metabolic syndrome in both humans (MetS) and horses (EMS) is characterized by a set of interrelated physiological, biochemical, clinical and metabolic factors, including insulin resistance (IR), abnormal glucose tolerance, hyperleptinemia and obesity.^1^, ^2^ However, recent data excluded obesity in horses as a *sine qua non* diagnostic factor.^3^, ^4^ Adipose tissue in both species is recognized as an active endocrine organ, responsible for the synthesis and secretion of several hormones controlling nutritional intake (leptin, angiotensin), insulin sensitivity and inflammatory mediators, eg tumour necrosis factor α (TNF‐α), resistin, visfatin, adiponectin and others.^5^ Importantly, abundant infiltration of adipose tissue by pro‐inflammatory (M1) macrophages and CD4^+^ T lymphocytes, combined with adipocytes’ hypertrophy, induces its dysfunction, characterized by increased IR, hypoxia and enhanced apoptosis.^6^, ^7^, ^8^ Furthermore, excessive accumulation of reactive oxygen species (ROS), nitric oxide (NO), protein kinase C activity, with a simultaneous decrease in superoxide dismutase (SOD) activity, which provides antioxidant defence, ultimately leads to the development of cardiovascular diseases in humans and can cause *laminitis* in horses.^9^, ^10^, ^11^ Additionally, a growing body of evidence suggests that in addition to inflammation, excessive oxidative stress (OS), ie ROS generated by mitochondria (MTs), plays a critical role in the development of obesity‐related diseases as well as degradation processes.^6^, ^12^ Moreover, ectopic accumulation of lipids promotes lipotoxicity, which in turn impairs cellular functions not only of adipocytes, but also of other adipose tissue components, causing IR, apoptosis and inflammation. Microenvironment, combined with OS and inflammation in adipose tissues of EMS horses, is recognized as one of the most important factors that contributes to accelerated senescence and ageing.^1^ Both inflammation and progressive ageing of adipose tissue are not without significance for adipose derived stem cells (ASCs) that reside within this tissue.

Adipose‐derived mesenchymal stromal stem cells are increasingly often recognized as a therapeutic source of stem cells and recently have been extensively used in veterinary practice.^13^ Clinical trials in humans have already been established for the intravenous administration of ASCs in autoimmune and inflammatory disorders, such as multiple sclerosis and arthritis.^14^ The growing interest in ASCs’ clinical applications results from their unique immunomodulatory and anti‐inflammatory effects as well as self‐renewal potential. ASCs express specific surface markers, including CD90^+^, CD105^+^ and CD44^+^, and they do not express CD45^−^. Moreover, ASCs have the ability to differentiate into adipocytes, myocytes, chondrocytes and osteoblasts, which underlines their potential utility in future cell‐based therapies. The pro‐regenerative properties of ASCs are explained by their paracrine and autocrine activities based on the secretion of membrane‐derived extracellular vesicles (ExMVs), which are known to play a critical role in intracellular signalling.^15^, ^16^ ExMVs were demonstrated to contain a broad range of growth factors, including vascular endothelial growth factors, fibroblast growth factors and transforming growth factor‐β—all of which are crucial in the treatment of MetS.^17^ Moreover, mesenchymal stem cells (MSCs) were shown to improve metabolic control in experimental models of type 2 diabetes (T2D), as measured by enhanced insulin secretion, improved insulin sensitivity and increased number of islet cells in the pancreas.^18^ Therefore, they are a promising tool also in the field of endocrinology.

Mitochondria play a pivotal role in energy metabolism, longevity and cell death. Moreover, recent studies have indicated that mitochondrial dynamics regulates tissue homeostasis and directs stem cell fate. Mitochondrial biogenesis was shown to be markedly induced during osteo‐ and adipogenic differentiation of MSCs, resulting in a high number of MT in differentiated cells. MTs are activated during osteogenic differentiation through an unknown mechanism, resulting in a bioenergetic switch. MSCs rely mainly on oxidative metabolism and contain a higher ATP content in comparison to undifferentiated counterparts. MTs are one of the major regulators of multipotency, and thus the physiological state of stem cells is closely related to the effectiveness of differentiation. Excessive accumulation of ROS induces cellular damage via protein and organelle oxidation. Previous studies have suggested that ROS accumulation strongly impairs osteogenic differentiation by directly affecting MTs and signalling pathways essential for bone development. ROS also affect MTs by disturbing their homeostasis, functionality and dynamics. In consequence, impaired organelles are not able to orchestrate proper progression of differentiation. Research has demonstrated that MT functions and metabolism need to be considerably enhanced upon osteogenic induction to fulfil high energy demand and facilitate biochemical reactions. However, under certain conditions, including diabetes and MetS, MSC MT are severely defective which strongly limits differentiation effectiveness.

Recent studies have shown that mitochondrial fusion proteins are up‐regulated in the early stages of adipo‐ and osteogenic differentiation, leading to MT elongation. On the other hand, enhanced expression of fission and autophagy proteins was observed during chondrogenesis. It is speculated that increased removal of MT during differentiation results in enhanced mitochondrial turnover. Thus, triggering autophagy promotes differentiation. Therefore, searching for chemicals capable of restoring MT functions is strongly desirable. RES is a well‐known natural, polyphenolic compound and autophagy activator. What is more, RES has been thoroughly documented as an immunomodulatory, anti‐inflammatory and antioxidant agent.^19^, ^21^, ^22^ It has been shown that RES has the ability to prolong lifespan, increase insulin sensitivity and protect against age‐related disorders, including T2D in mice fed a high‐fat diet.^23^ Moreover, it was found that RES activated AMPK/PGC‐1α signalling, improved mitochondrial biogenesis and dynamics, reduced IGF‐1 levels and inhibited apoptosis by stimulating autophagy in a mouse model of diabetic nephropathy.^22^ Finally, a study conducted by Shakibaei et al showed that RES promoted osteogenic differentiation in human and mouse MSCs through Sirt1/Runx2 activation. Interesting data regarding the involvement of RES in mediating MT removal have been published recently.^23^ It was shown that RES induced Pink and Parkin expression in senescent cardiomyocytes to degrade impaired MT. It has been speculated that the activation of these proteins may be a potential mechanism by which RES reverses the impairment of aged cells. ASC senescence and ageing, which is associated with DNA methylation, can be diminished by the application of DNA methyltransferase (DNMT) inhibitors, such as 5‐azacitidine (5‐AZA), which inhibits the methylation pattern of specific gene regions, while activating associated genes. 5‐AZA is chemically an analogue of cytidine, commonly used in the treatment of acute myelogenous leukaemia.^24^ However, it was shown in human‐aged ASCs that 5‐AZA reversed their aged phenotype, increased proliferative activity and improved osteogenic differentiation potential, while it decreased the accumulation of OS factors and DNA methylation status.^25^ Moreover, a study conducted by Seeliger et al^26^ revealed that AZA improved metabolic and enzymatic activity in hepatocyte‐like cells.

Previous studies have shown that ASCs isolated from EMS horses (ASC_EMS_) suffered from reduced proliferative activity, progressive apoptosis, ageing and senescence, which eventually impaired their multipotency and osteogenic differentiation potential.^27^, ^28^, ^29^ It was shown that ASCs_EMS_ exhibited reduced secretion of extracellular microvesicels (ExMVs) rich in bone morphogenetic protein 2 (BMP‐2), collagen type II (COll‐2) or osteocalcin (OCN)—all fundamental for ontogenesis. Deterioration of differentiation potential was caused by dysfunction of mitochondrial metabolism and dynamics. ASCs_EMS_ were characterized by down‐regulation of genes related to selective mitophagy and mitochondrial biogenesis, ie PINK, PARKIN, PGC1α and PDK4. Accumulation of damaged MT triggers their segregation from the mitochondrial network and targets these organelles for autophagic degradation in a process that requires Parkin‐dependent ubiquitination of mitochondrial proteins. *Ex vivo* induction of the proper elimination of dysfunctional MT in the course of osteogenic differentiation may be essential for cell survival, extracellular matrix formation and maintenance of “stemness.” Therefore, the strategy to search for pharmaceutical intervention that would improve mitochondrial metabolism and dynamics, combined with reduced senescence, apoptosis, OS and ageing, during osteogenic differentiation in ASCs_EMS_ seems to be reasonable. What is more, recent discoveries underlined the requirement for ex vivo ASC_EMS_ “recovery,” as it has been demonstrated that allogeneic MSCs may affect the immune response in donor animals, and that allogeneic administration of ASCs is not as safe as previously thought.

The aim of the present study was to investigate whether *ex vivo* treatment of ASC_EMS_ with a combination of AZA/RES would reverse aged phenotype and promote osteogenic differentiation potential in ASC_EMS_. For this purpose, mitochondrial metabolism and dynamics, autophagy, OS and senescence of ASC_EMS_ cultured under osteogenic differentiation conditions were investigated. Finally, we analysed protein and mRNA levels of osteogenesis master regulators, including Runx, BMP‐2, OCN and osteopontin.

## 2 MATERIALS AND METHODS

All reagents used in this experiment were purchased from Sigma‐Aldrich (Poland), unless indicated otherwise.

### 2.1 Qualification of horses

Animals were age‐matched (mixed sex, 9‐14 years; mean ± SD, 11.2 ± 1.7 years) and assigned into 2 groups: EMS (n = 5, 2 female, 3 male) and healthy horses (n = 5, 2 female, 3 male). Detailed characterization of animals used in this experiment is shown in Table 1. Qualification of animals was based on (*i*) extensive interviews with owners, (*ii*) measurement of body weight, (*iii*) estimation of body condition score (BCS) and cresty neck scoring system (CNS), (*iv*) palpation and visual assessment of the hoof capsule, (*v*) X‐ray examination, (*vi*) resting insulin levels, (*vii*) combined glucose‐insulin test (CGIT) and (*viii*) LEP concentration as described previously.^7^

**Table 1:** Criteria for horses’ classification

		Bw (kg)	BCS [1‐9]	CNS [1‐5]	Fasting insulin (mU/mL)	LEP (ng/mL)	CGIT:GLU in 45 min (mg/dL)
Healthy (CTRL)	Mean ± SD	633.6 ± 15.9	6.6 ± 0.5	1.8 ± 0.4	11.2 ± 2.6	3 ± 0.8	76.6 ± 8
EMS	Mean ± SD	731.4 ± 21.2	8.6 ± 0.5	3.6 ± 0.5	77.8 ± 12.6	7.6 ± 1.5	139.4 ± 4.6

BCS, body condition score; Bw, body weight; CGIT, combined glucose‐insulin test; CNS, cresty neck score; GLU, glucose; LEP, leptin; n, negative test results; p, positive test results; SD, standard deviation.

### 2.2 Cell isolation

Approximately, 2 g of subcutaneous adipose tissue was harvested from the horses’ tail base following the standard surgical procedure and ethical standards. Next, tissue samples were placed in sterile Hank's balanced salt solution (HBSS) and transported immediately to the laboratory. Cells were isolated under aseptic conditions following the previously described protocol by Marycz et al^29^ Briefly, specimens were extensively washed 3 times with HBSS, cut into small pieces and minced. Tissue was then digested in collagenase type I solution (1 mg/mL) for 40 minutes at 37°C. Following digestion, samples were centrifuged (1200×*g*, 10 minutes) and the supernatant was discarded. Remaining cell pellets were re‐suspended in culture medium and transferred to a culture flask. In order to perform the experiments, cells were passaged 3 times.

### 2.3 Cell culture

During the experiment, cells were cultured under aseptic and constant conditions in an incubator (37°C, 5% CO_2_ and 95% humidity). Culture medium consisted of DMEM with 4500 mg/L glucose supplemented with 10% fetal bovine serum (FBS) and 1% of penicillin‐streptomycin (PS). Culture medium was changed every 3 days. Cells were passaged after reaching 90% confluence using trypsin solution (TrypLE™ Express; Life Technologies).

### 2.4 Immunophenotyping and multipotency assay

The expression of the following surface antigens: CD44, CD45, CD90 and CD105 was investigated in isolated cells. The purity of ASCs was assessed using flow cytometry (BD FACSCalibur; Becton Dickinson, Franklin Lanes, NJ, USA). In order to perform the analysis, cells were detached from the dishes using TrypLE™ Express. Cell suspension was incubated at 4°C for 20 minutes with specific primary antibodies at the dilution of 1:500 (anti‐CD45; Novus Biologicals, Littleton, CO, USA, NB1006590APC; anti‐CD90, ab225; Abcam, Cambridge, UK; anti‐CD44, R&D Systems, Minneapolis, MN, USA, MAB5449). Cells were washed 3 times by centrifugation at 400× *g* for 5 minutes and re‐suspend in fluorochrome‐labelled secondary antibodies (Alexa Fluor 488, ab150113; Abcam). At least 10 000 stained cells were acquired and analysed by FACS Calibur flow cytometer. The samples were analysed using CellQuest Pro software (Becton Dickinson).

In order to confirm the multipotency of the isolated cells, they were differentiated into adipogenic, chondrogenic and osteogenic lineage. Prior the experiment, cells were maintained in 24‐well plates and seeded at an initial concentration of 1 × 10^4^ cells per well. Cells were cultured in StemPro adipogenesis, StemPro chondrogenesis and StemPro osteogenesis differentiation kits (Life Technologies) in accordance with the manufacturer's instructions. Cells were propagated for 11 days and media were changed every 2 days. In order to confirm the differentiation process, cells were fixed with 4% paraformaldehyde (PFA) and subjected to proper staining. Intracellular lipid droplets formed during adipogenesis were stained with Oil Red O, while an extracellular mineralized matrix formed in the course of osteogenesis was stained with Alizarin Red. After chondrogenic differentiation, formation of proteoglycans was visualized with Safranin O. Cells were observed under an inverted microscope (AxioObserverA1; Zeiss, Oberkochen, Germany) and pictures were taken using Cannon PowerShot digital camera.

### 2.5 Proliferation rate

Growth kinetics of ASCs was examined using a resazurin assay kit (TOX8), following the manufacturer's instructions. To perform the assay, cells were plated in 24‐well plates at an initial concentration of 2 × 10^4^ per well. Culture media were replaced with media containing 10% of resazurin dye and cells were incubated in a CO_2_ incubator, 37°C for 2 hours. Next, supernatants were transferred to 96‐well plates and subjected to spectrophotometric assay (Epoch; BioTek). The absorbance of the supernatants was measured at a wavelength of 600 nm for resazurin, and 690 nm reference wavelength. Population doubling time (PDT) was assessed with the support of a PDT online calculator (http://www.doubling-time.com/compute.php).

Moreover, DNA synthesis was investigated by measuring the incorporation of 5‐bromo‐2‐deoxyuridine (BrdU) into cellular DNA. Test was performed with BrdU Cell Proliferation ELISA Kit (Abcam) in accordance with the manufacturer's protocol. Briefly, cells were incubated with BrdU overnight at 37°C and fixed following DNA denaturation. Incorporation of BrdU was evaluated by incubation with anti‐BrdU monoclonal antibody. IgG conjugated with horseradish peroxidase was used as a secondary antibody. Colour reaction was developed using 3,3,5,5‐tetramethylbenzidine (TMB). Signal intensity was measured at a wavelength of 450/550 nm (Epoch; BioTek).

### 2.6 SEM, TEM and FIB‐SEM analysis

Detailed morphology of cells was evaluated using scanning electron microscope (Zeiss EVO LS15). First, cells were fixed in 4% PFA and washed with HBSS 3 times. Next, specimens were dehydrated in a graded ethanol series (from 50% to 100%). Air‐dried samples were sputtered with gold (ScanCoat 6; Oxford), placed in a microscope chamber and observed using a SE1 detector, at 10 kV of filament's tension. SEM was equipped with a BRUCKER energy dispersive X‐ray system (SEM/EDX), which allowed to analyse the distribution of the elemental composition of the samples. Using SEM/EDX, calcium and phosphorus concentration was established. The quantax detector (Brüker) with 10 kV of filament tension was used to perform a line scan analysis of randomly selected cells. The obtained values were presented as weight percentage (wt%). Moreover, we estimated the number and size of bone nodules in each group.

In order to perform transmission electron microscopy (TEM) analysis, cells were fixed in 2.5% glutaraldehyde at 4°C. After fixation, cells were centrifuged at 2000 *g* for 10 minutes and rinsed with PBS for 30 minutes. Then samples were incubated with 1% osmium tetroxide in HBSS for 2 hours, washed and centrifuged again. Further, samples were dehydrated in graded acetone series (30%‐100%), embedded using Agar Low Viscosity Resin Kit (Agar Scientific Ltd., Essex, UK). Ultrathin sections (80 nm) were collected on copper grids. Uranyl acetate and lead citrate were used for contrasting. The observations were carried out using Auriga60 Zeiss STEM, at 20 kV filament tension.

Cells were analysed using FIB‐SEM using a previously described method.^27^ Briefly, samples were fixed with 2.5% glutaraldehyde in 0.1 mol/L cacodylate buffer and incubated on ice in 2% osmium tetroxide/1.5% potassium ferricyanide for 1 hour. After incubation, cells were dehydrated in graded ethanol series and infiltrated with resin (Agar Low Viscosity Resin Kit, Agar, UK). Resin blocks with recovered cell‐containing surfaces were mounted on microscope stubs with a carbon tape, maintaining the horizontal position of the recovered surfaces, and sputtered with 20 nm layer of gold (Leica EM ACE600). Next, specimens were placed in a field‐emission cross‐beam electron/ion microscope (Zeiss Auriga 60). Prior to focused ion beam (FIB) milling, the areas containing chosen cells were covered with ~50 nm protective layer of platinum. The imaging was performed using SE2 detector at 2 kV of electron beam voltage. Using obtained photographs, mitochondrial net was further reconstructed in Imaris Software.

### 2.7 Confocal imaging

Prior MT visualization, cells were incubated with MitoRed dye (1:1000) in 37°C for 30 minutes, fixed with PFA and rinsed with HBSS. Nuclei were counterstained with diamidino‐2‐phenylindole (DAPI; 1:1000 in HBSS) for 5 minutes. For immunofluorescence, cells were fixed in 4% PFA and permeabilized with 0.5% Triton X‐100. Non‐specific binding sites were blocked with blocking buffer (10% Goat Serum, 0.2% Tween‐20 in HBSS) for 45 minutes. Cells were then incubated overnight at 4°C with primary antibodies against LAMP2 (Abcam) diluted 1:500 in HBSS containing 1% Goat Serum and 0.2% Tween‐20. After washing, samples were incubated for 1 hour with goat anti‐mouse secondary antibodies conjugated with atto‐488 (dilution 1:1000; Abcam). Nuclei were counterstained with DAPI. Specimens were observed and photographed using confocal microscope (Observer Z1 Confocal Spinning Disc V.2 Zeiss with live imaging chamber) and analysed using ImageJ software. Based on representative photographs, mitochondrial net was visualized in microP software.^30^

### 2.8 Quantification of ALP levels

In order to examine extracellular ALP activity, an Alkaline Phosphatase Colorimetric Assay Kit (Abcam) was used in accordance with the manufacturer's protocol. In the assay, the p‐nitrophenyl phosphate was used as a phosphatase substrate. Obtained product was measured at 405 nm wavelength (Epoch; BioTek). The amount of pNP was obtained by sample readings applied to a standard curve. ALP activity was calculated using the following formula: ALP activity (U/mL) = A/V/T (where A—pNP amount; V—volume of sample added to well (mL); T—reaction time).

### 2.9 Oxidative stress factors and senescence

Nitric oxide concentration was assessed using reagent kit (Life Technologies) while SOD activity was measured using a SOD Assay kit (Sigma‐Aldrich). ROS were estimated with an H2DCF‐DA (Life Technologies).

The presence of senescence associated β‐galactosidase (β‐gal) in cells was visualized using a commercial kit (Senescence Cells Histochemical Staining Kit; Sigma‐Aldrich) according to the manufacturer's protocol. The percentage of cells expressing β‐gal (stained blue) in regard to β‐gal negative cells was calculated. The number of viable and dead cells was established with Cellstain Double Staining Kit (Sigma‐Aldrich). Viable cells were stained with Calcein‐AM whereas dead cells’ nuclei were with Propidium Iodide.

To assess the mitochondrial membrane's potential, the cell pellets were treated with 1 mmol/L JC‐1 reagent (Life Technologies) in accordance with the manufacturer’ instructions. Obtained results were analysed with CellQuest Pro Software.

### 2.10 siRNA transfection

To knock down PARKIN expression in ASC_EMS_, a PARKIN small interfering RNA (siRNA) construct was purchased and tested for knockdown efficiency. ASC were seeded at an initial density of 1 × 10^5^ per cm^2^ and incubated with 8 μL of 20 μmol/L siRNA in 100 μL of OPTIMEM (Life Technologies) with 10 μL of Lipofectamine 2000 (Life Technologies). After 72 hours, total RNA was isolated from cells using TRI Reagent.

### 2.11 Reverse‐transcription polymerase chain reaction (RT‐PCR)

In order to isolate total RNA, cells were homogenized with 1 mL of TRI Reagent. Total RNA was isolated according to method described by Chomczynski and Sacchi.^31^ The quantity and quality of received genetic material were estimated using a nanospectrophotometer (Epoch; Biotek). Genomic DNA was digested using DNase I, RNAase‐free (Life Technologies) while complementary DNA (cDNA) synthesis was performed using RevertAid RT Reverse Transcription Kit (Life Technologies). Each reaction contained 150 ng of total RNA. Both procedures were carried out following the manufacturer’s protocol using T100 Thermal Cycler (Bio‐Rad) as described elsewhere.^32^

The qRT‐PCR reactions were performed using SensiFast SYBR & Fluorescein Kit (Bioline,) and a CFX ConnectTM Real‐Time PCR Detection System (Bio‐Rad). Each reaction mixture contained 2 μL of cDNA in a total volume of 20 μL, while the primers’ concentration was 0.5 μmol/L per sample. Sequences of the primers used in the amplification are listed in Table 2.

**Table 2:** Sequences of primers used in RT‐PCR

Gene	Primer	Sequence 5′‐3′	Amplicon length (bp)
LC3	F:	TTACTGCTTTGCTCTGCCAC	213
	R:	AGCTGCTTCTCCCCCTTGT	
Beclin‐3	F:	GATGCGTTATGCCCAGATGC	147
	R:	ATCCAGCGAACACTCTTGGG	
LAMP‐2	F:	GCACCCCTGGGAAGTTCTTA	139
	R:	TTCGAGGATCTGTGCCAATCA	
GAPDH	F:	GATGCCCCAATGTTTGTGA	250
	R:	AAGCAGGGATGATGTTCTGG	
CHOP	F:	AGCCAAAATCAGAGCCGGAA	272
	R:	GGGGTCAAGAGTGGTGAAGG	
PERK	F:	GTGACTGCAATGGACCAGGA	283
	R:	TCACGTGCTCACGAGGATATT	
PINK	F:	GCACAATGAGCCAGGAGCTA	298
	R:	GGGGTATTCACGCGAAGGTA	
PARKIN	F:	TCCCAGTGGAGGTCGATTCT	218
	R:	CCCTCCAGGTGTGTTCGTTT	
FIS	F:	GGTGCGAAGCAAGTACAACG	118
	R:	GTTGCCCACAGCCAGATAGA	
MFN‐1	F:	AAGTGGCATTTTTCGGCAGG	217
	R:	TCCATATGAAGGGCATGGGC	
p53	F:	TACTCCCCTGCCCTCAACAA	252
	R:	AGGAATCAGGGCCTTGAGGA	
p21	F:	GAAGAGAAACCCCCAGCTCC	241
	R:	TGACTGCATCAAACCCCACA	
Cas‐9	F:	TCCTACTCCACCTTCCCAGG	150
	R:	CTCCGAAACAGCGTGAGCTA	
p62 (SQSTM)	F:	CATCGGAGGATCCCAGTGTG	207
	R:	CCGGTTTGTTAGGGTCGGAA	
TET 2	F:	ATCCTGATCCTGGTGTGGGA	143
	R:	CCTTGACAGGCACAGGTTCT	
TET 3	F:	CAGCCTGCATGGACTTCTGT	188
	R:	GTTCTCCTCACTGCCGAACT	
DNMT‐1	F:	GGCGAAAGCGGACAATTCTG	90
	R:	AGCGGTCTAGCAACTGGTTC	
Cas‐3	F:	GGCAGACTTCCTGTATGCGT	167
	R:	CCATGGCTACCTTGCGGTTA	
PGC1‐α	F:	GGCCTTCTAAACGTGGGACA	135
	R:	CCGGAGGTCTGCCATTTTCT	
OPN	F:	CATCGCCTATGCCCTTCCAG	217
	R:	TGTGTGGTCATGGCTTTCGT	
RUNX‐2	F:	CCAAGTGGCAAGGTTCAACG	165
	R:	TGTCTGTGCCTTCTGGGTTC	
Coll‐I	F:	GAAACTATCAATGGTGGTACCA	265
	R:	AGCAGCCATCTACAAGAACAGT	
BMP‐2	F:	CGTCCTGAGCGAGTTCGAGT	165
	R:	CGCCGGGTTGTTTTTCCACT	
IL‐10	F:	TGTTGTTGAACGGGTCCCTG	242
	R:	ACTCTTCACCTGCTCCACTG	
iNOS	F:	GACAAGCTGCATGTGACATC	325
	R:	GCTGGTAGGTTCCTGTTGTT	
TNF‐α	F:	ACAGAAAGCATGATCCGCGA	295
	R:	CTTGGTGGTTTGCTACGACG	
IL‐6	F:	GAGGATACCACTCCCAACAGACC	141
	R:	AAGTGCATCATCGTTGTTCATACA	
miR‐451		AAACCGTTACCATTACTGTGTT	

Beclin, beclin 1, autophagy related (BECN1); BMP‐2, Bone morphogenetic protein 2; Cas‐3, caspase‐3; CHOP, DNA damage‐inducible transcript 3; Coll‐1, collagen type 1; DNMT‐1, DNA (cytosine‐5)‐methyltransferase 1; FIS, mitochondrial fission 1 molecule; GADPH, Glyceraldehyde‐3‐Phosphate Dehydrogenase; IL‐10, interleukin 10; IL‐6, interleukin 6; iNOS, nitric oxide synthase; LAMP2, lysosomal‐associated membrane protein 2; LC3, microtubule‐associated protein 1 light chain 3 beta (MAP1LC3B); MFN1, mitofusin 1; OPN, osteopontin; p21, Cyclin‐Dependent Kinase Inhibitor 1A, Cas‐9, caspase‐9; p53, tumour suppressor p53; p62, Sequestosome‐1; PARKIN, parkin RBR E3 ubiquitin protein ligase (PARK2); PERK, PRKR‐like endoplasmic reticulum kinase; PGC1‐α, Peroxisome proliferator‐activated receptor gamma coactivator 1‐α; PINK, PTEN‐induced putative kinase 1 (PINK1); RUNX‐2, Runt‐related transcription factor 2; TET 2, Tet methylcytosine dioxygenase 2; TET 3, Tet Methylcytosine Dioxygenase 3; TNF‐α, tumour necrosis factor α.

To determine miRNA expression, 500 ng of RNA was reverse‐transcribed using a Mir‐X miRNA First‐Strand Synthesis Kit (Takara Bio Europe) and then subjected for quantitative PCR (final volume 20 μL) with SYBR Advantage qPCR Premix (Takara Bio Europe). The reaction included initial denaturation at 95°C for 10 seconds, followed by 55 cycles of 95°C for 5 seconds and annealing temperature 60°C for 20 seconds with a single fluorescence measurement.

The average fold change in the gene expression of experimental cultures was compared with control cultures and calculated by the 2^−ΔΔ^ *C*_t_ method in relation to the housekeeping gene—GAPDH and U6snRNA for miRNA quantification. Furthermore, the ratio of MFN/FIS was calculated by dividing MFN and FIS relative expression (2^−ΔΔ^ *C*_t_).

### 2.12 Co‐culture of ASC with RAW 264.7

RAW 264.7 were counted, adjusted to a density of 1 × 10^6^ cells/mL and seeded onto a 24‐well plate in a volume of 500 μL/well. After 18 hours, non‐adherent cells were removed by a gentle wash with warm HBSS. Next, LPS was added to the culture media at a concentration of 1 μg/mL and the experiment was continued for another 24 hours. At the same time, ASC in the amount of 4 × 10^4^ was added to culture wells. After 24 hours of co‐culture, media were collected for analysis of the macrophages’ secretory activity, and the cells were lysed by adding TRI Reagent.

### 2.13 ELISA assays

The concentration of TNF‐α and OPN protein in cell‐free culture supernatants was determined by solid‐phase ELISA using the Mouse TNF‐α Quantikine ELISA Kit (R&D Systems) and Horse OPN ELISA Kit (MyBioSource, San Diego, CA, USA), respectively. Procedure and calculations were carried out in accordance with the manufacturer's instruction. The absorbance was measured with a 96‐well microplate reader (Epoch; BioTek) at 450 nm.

### 2.14 Western blotting

Cells were detached from culture dishes and homogenized in RIPA buffer plus protease inhibitor cocktail. The lysates were centrifuged at 4°C for 20 minutes (14 000 *g*) and supernatants were transferred to new tubes. Thirty micrograms of protein were used for each sample. SDS‐PAGE was performed at 100 V for 90 minutes in Tris/glycine/SDS buffer. Proteins were transferred onto a polyvinylidene difluoride membrane (Bio‐Rad) using a transfer apparatus at 100 V for 1 hour at 4°C in Tris/glycine buffer. After transfer, the membrane was washed with Tris/NaCl/Tween buffer (TBST) and blocked overnight at 4°C with 5% non‐fat milk in TBST. Next, the membrane was washed with TBST and incubated with primary antibody for 2 hours: βAKT (Sigma‐Aldrich, A5441), PARKIN (Novus, NB100‐91921), LAMP‐2 (Abcam, ab15580), TET‐2 (Abcam, ab124297), MFN‐1 (Biorbyt, orb11040,) and MFF (Biorbyt orb325479) at a dilution of 1:500. After washing the membrane, a solution of appropriate secondary antibody conjugated with ALP was applied. After 2‐hours incubation, the membrane was washed again with TBST and incubated with BCIP^®^/NBT‐Purple Liquid Substrate for 15 minutes. The reaction was stopped by washing the membrane with water.

### 2.15 Evaluation of 5‐methylocytosine (5‐mC)‐ and histone H3‐positive cells

All flow cytometry analyses were performed after 24 hours of the osteogenic differentiation. To evaluate the 5‐mC‐ and histone H3‐positive cells, ASCs were detached from culture dishes and centrifuged at 350× *g* for 5 minutes following fixation with 4% ice‐cold PFA. The cells were washed extensively with HBSS and incubated with 0.1% Tween diluted in HBSS for 20 minutes. Biological material was incubated with anti‐5mC antibody (Abcam, ab73938) and anti‐histone H3 (Abcam, ab8898) solution supplemented with 10% goat serum for 30 minutes at 22°C. Afterwards, the cells were incubated with Alexa 488 goat anti‐mouse secondary antibodies (1:500, Alexa Fluor 488; Abcam) for 30 minutes at 22°C.

### 2.16 Statistical analysis

All experiments were performed at least in 3 replicates. Differences between experimental groups were estimated using unpaired student's *t* test. Statistical analysis was conducted with GraphPad Prism 5 Software (La Jolla, USA). Differences with probability of *P *<* *.05 were considered significant. Statistical significance is indicated as asterisk (*) when comparing the result to ASC_CTRL_, and as hashtag (#) when comparing to ASC_EMS_.

## 3 RESULTS

### 3.1 Immunophenotyping and multipotency assay

**Figure 1:**
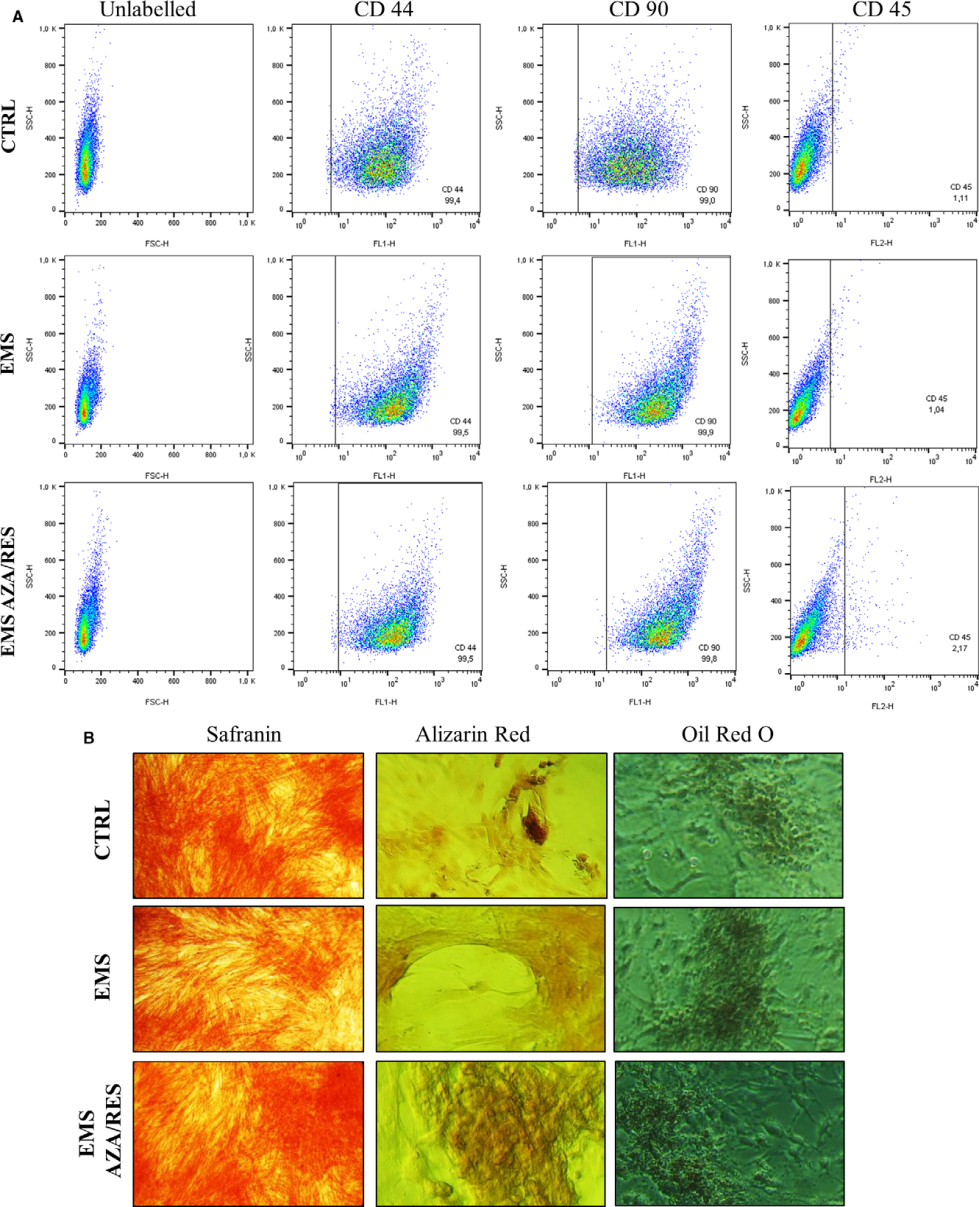
Immunophenotyping and multipotency assay. In order to confirm the multipotent character of isolated cells, they were analysed for CD44, CD45 and CD90 surface antigens’ expression with flow cytometer (A). Furthermore, cells were differentiated into chondrogenic, osteogenic and adipogenic lineage (B). Effectiveness of differentiation was confirmed by specific staining

In order to characterize surface marker expression, cells were analysed by flow cytometry. Both ASC cell populations (ASC_CTRL_ and ASC_EMS_) fulfilled the requirements of the International Society for Cellular Therapy in assuring MSC identity by displaying plastic adherent growth and exhibiting high CD44, CD90 expression and lack of CD45 hematopoietic marker (Figure 1A). Additionally, multipotent characteristics of ASCs were confirmed by positive results of differentiation into osteoblast, chondrocytes and adipocytes in vitro, as demonstrated by specific lineage staining (Figure 1B).

### 3.2 Proliferation rate of AZA/RES‐treated cells in control and osteogenic conditions

**Figure 2:**
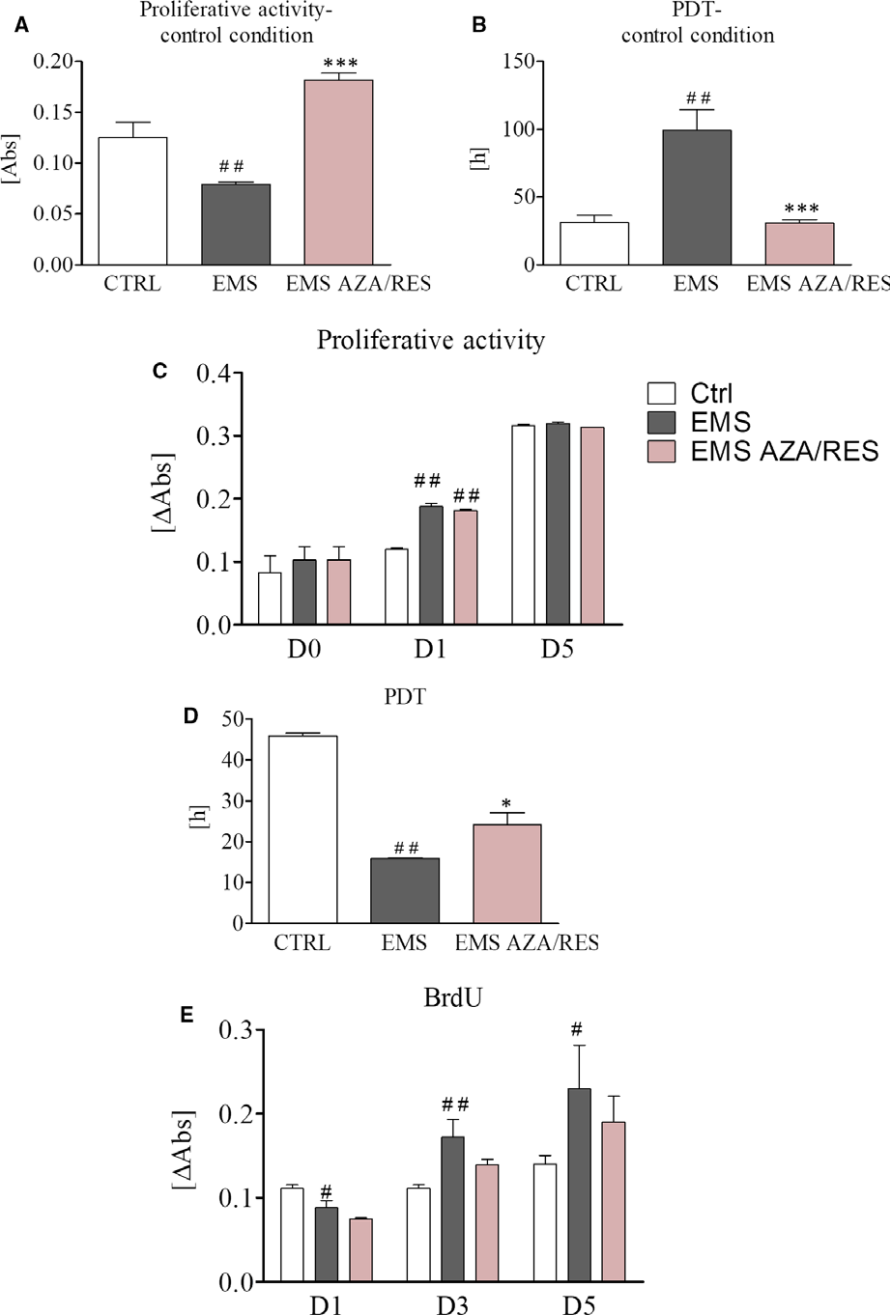
Proliferation of cells pre‐treated with AZA/RES in control and osteogenic conditions. Cells were cultured in standard medium supplemented with AZA/RES for 24 h and subjected to alamar blue assay in order to investigate their proliferation rate (A). Untreated cells served as a control group (CTRL and EMS). Furthermore, PDT was assessed as well (B). Proliferation rate (C) PDT (D) and BrdU (E) established for cells cultured in osteogenic medium. Results expressed as mean ± SD. Statistical significance indicated as asterisk (*) when comparing the result to ASC_CTRL_, and as hashtag (#) when comparing to ASC_EMS__._ #*P *<* *.05, ##*P *<* *.01, *P <* *.05, ***P <* *.001

In order to perform the experiments, cells were pre‐treated with AZA/RES for 24 hours (indicated as day 1 in case of osteogenic differentiation). In control medium, after 24 hours of propagation in the presence of AZA/RES, significantly increased proliferation rate was observed in the experimental group in comparison to untreated cells (Figure 2A, *P* < .001). Those results were further supported by the analysis of PDT. ASC_EMS_ displayed increased PDT, while in the experimental group, time required to double cell number was diminished and comparable to control, healthy cells (Figure 2B, *P* < .001). Proliferation rate during osteogenesis was assessed during 3 distinct time points. D0 refers to day after cell seeding, d1 after 24 hours of AZA/RES treatment and D5 to fifth day of osteogenic differentiation. Statistically significant differences in the proliferation rate during differentiation were only noted at day D1, as both ASC_EMS_ and ASC_EMS AZA/RES_ displayed increased proliferation in comparison to the control group (Figure 2C, *P* < .01). PDT in ASC_EMS_ was down‐regulated in comparison to control cells (Figure 2D, *P* < .01) and ASC_EMS AZA/RES_ (*P *<* *.05). Furthermore, during osteogenic differentiation, incorporation of BrdU was investigated in counterpart time points. No differences were noted between ASC_EMS_ and ASC_EMS AZA/RES_. However, incorporation of BrdU in ASC_EMS_ at D1 decreased in comparison to ASC_CTRL_ while increased at D3 and D5 (Figure 2E, *P* < .01 and *P *<* *.05, respectively).

### 3.3 Evaluation of osteogenesis effectiveness

**Figure 3:**
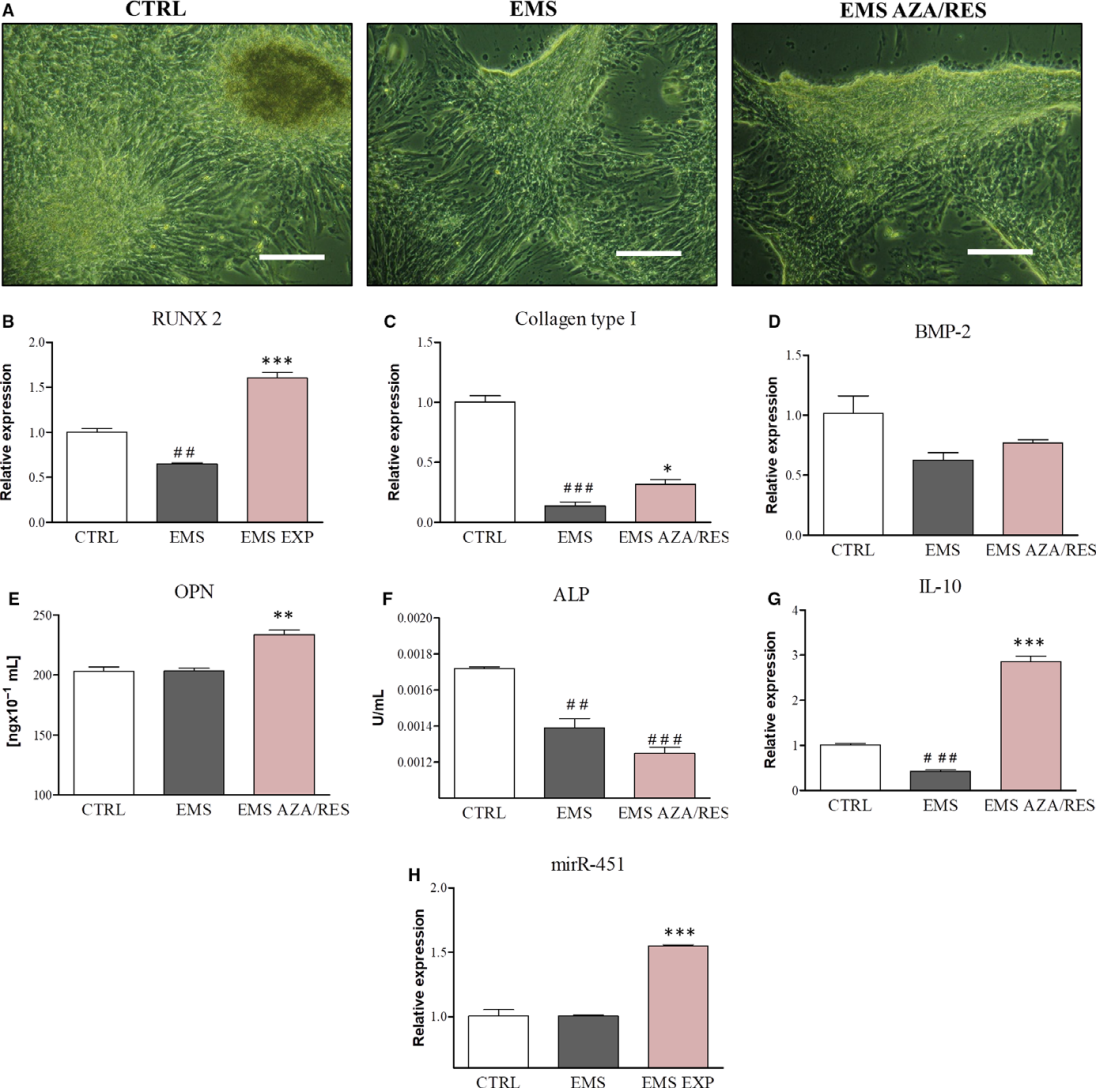
Evaluation of osteogenesis effectiveness. After day 5 of osteogenesis, cells were captured using inverted, light microscope (A) and the formation of a mineralized matrix was visualized. Using RT‐PCR we analysed the expression of osteogenic‐related genes including RUNX 2 (B), collagen I (C) and BMP‐2 (D). Secretion of OPN was tested with ELISA assay (E). ALP activity (F) was reduced in both EMS groups as indicated (F). As IL‐10 plays a role in osteogenic differentiation, we evaluated its amount with ELISA (G). Furthermore, using RTR‐PCR the expression of miR‐451 was tested (H). Results expressed as mean ± SD. Statistical significance indicated as asterisk (*) when comparing the result to ASC_EMS_, and as hashtag (#) when comparing to ASC_CTRL__._ ##*P *<* *.01, ###*P *<* *.001, *P <* *.05, **P <* *.01, ***P <* *.001. Magnification ×100, scale bar: 250 μm

Photographs taken with light microscope indicated the robust formation of mineralized matrix in ASC_CTRL_. On the contrary, ASC_EMS_ were characterized by an increased number of undifferentiated cells. However, treatment with AZA/RES markedly enhanced the differentiation process (Figure 3A). Using RT‐PCR we evaluated the expression of osteogenic‐related genes. RUNX 2 mRNA was down‐regulated in ASC_EMS_ in comparison to the control group (Figure 3B, *P* < .01); however, AZA/RES treatment significantly enhanced its expression (*P *<* *.001). Collagen I expression was diminished in ASC_EMS_ (Figure 3C, *P* < .001), although after AZA/RES treatment its levels increased (*P *<* *.05). No statistically significant differences were found in the expression of BMP‐2 between groups. ASC_EMS_ preconditioned with AZA/RES significantly enhanced the secretion of OPN (Figure 3E, *P* < .01). However, both ASC_EMS_ and ASC_EMS AZA/RES_ displayed diminished ALP activity in comparison to control cells (Figure 3F, *P* < .01 and *P *<* *.001 respectively). IL‐10 secretion by MSC is associated with the promotion of osteoblastic differentiation. We found decreased synthesis of IL‐10 in ASC_EMS_ (Figure 3G, *P* < .001); however, its production was significantly increased in AZA/RES group (*P *<* *.001). Furthermore, the expression of miR‐451 was significantly up‐regulated after AZA/RES treatment (Figure 3H, *P* < .001).

### 3.4 Assessment of bone mineralization

**Figure 4:**
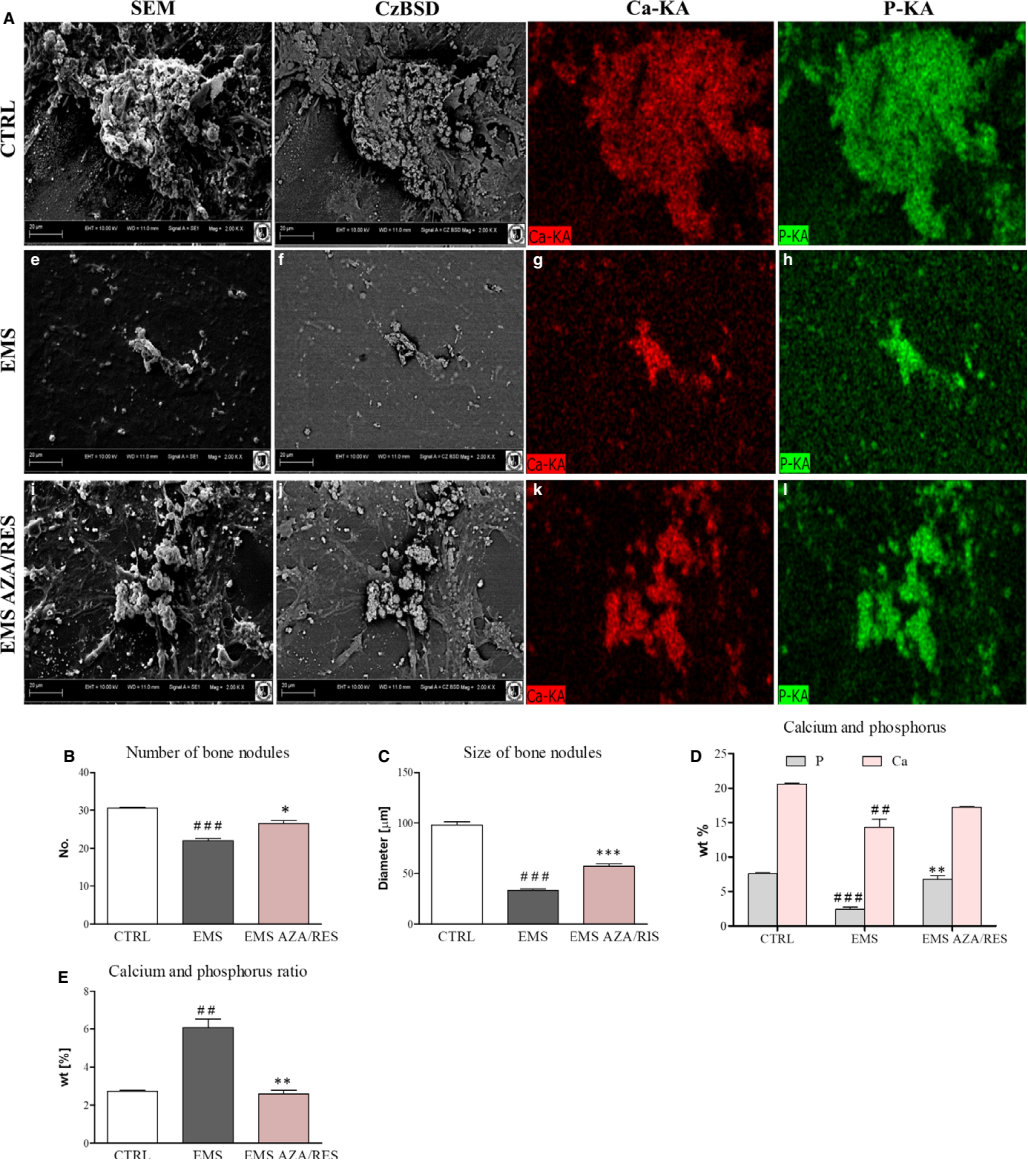
Matrix mineralization in cells cultured in control and AZA/RES conditions. Using SEM we visualized the detailed morphology of newly formed matrix, levels of organic material within bone nodules (CzBSD), Ca and P concentration (A). Data obtained from SEM were further quantified as mean number of bone nodules (B), their size (C), amount of Ca/P (D) as well as Ca/P ratio were calculated (E). Results expressed as mean ± SD. Statistical significance indicated as asterisk (*) when comparing the result to ASC_EMS_, and as hashtag (#) when comparing to ASC_CTRL_. ##*P *<* *.01, ###*P *<* *.001, *P <* *.05, **P <* *.01, ***P <* *.001

Using SEM with EDX and CzBSD detectors, we evaluated the effectiveness of mineralized matrix formation (Figure 4A). CzBSD detector allowed for the visualization of organic compounds (brighter signal refers to more organic compounds), whereas EDX was used for the evaluation of Ca and P content. Results obtained as photographs were further quantified. The number of bone nodules was decreased in ASC_EMS_ in comparison to the control group (Figure 4B, *P* < .001); however, AZA/RES treatment significantly increased nodules’ number (*P *<* *.05). Analogous phenomenon was observed in nodules’ size (Figure 4C). Ca and P amounts were significantly down‐regulated in ASC_EMS_ (Figure 4D, *P* < .001 and *P *<* *.01, respectively). However, Ca levels were increased in ASC_EMS AZA/RES_ (*P *<* *.01). Notably, AZA/RES treatment did not affect P concentration. Furthermore, the ratio of Ca and P was calculated (Figure 4E). Robust accumulation of Ca in comparison to P was observed in ASC_EMS_ (*P *<* *.01), while AZA/RES treatment resulted in a decreased ratio of Ca and P (*P *<* *.01).

### 3.5 Accumulation of oxidative stress factors

**Figure 5:**
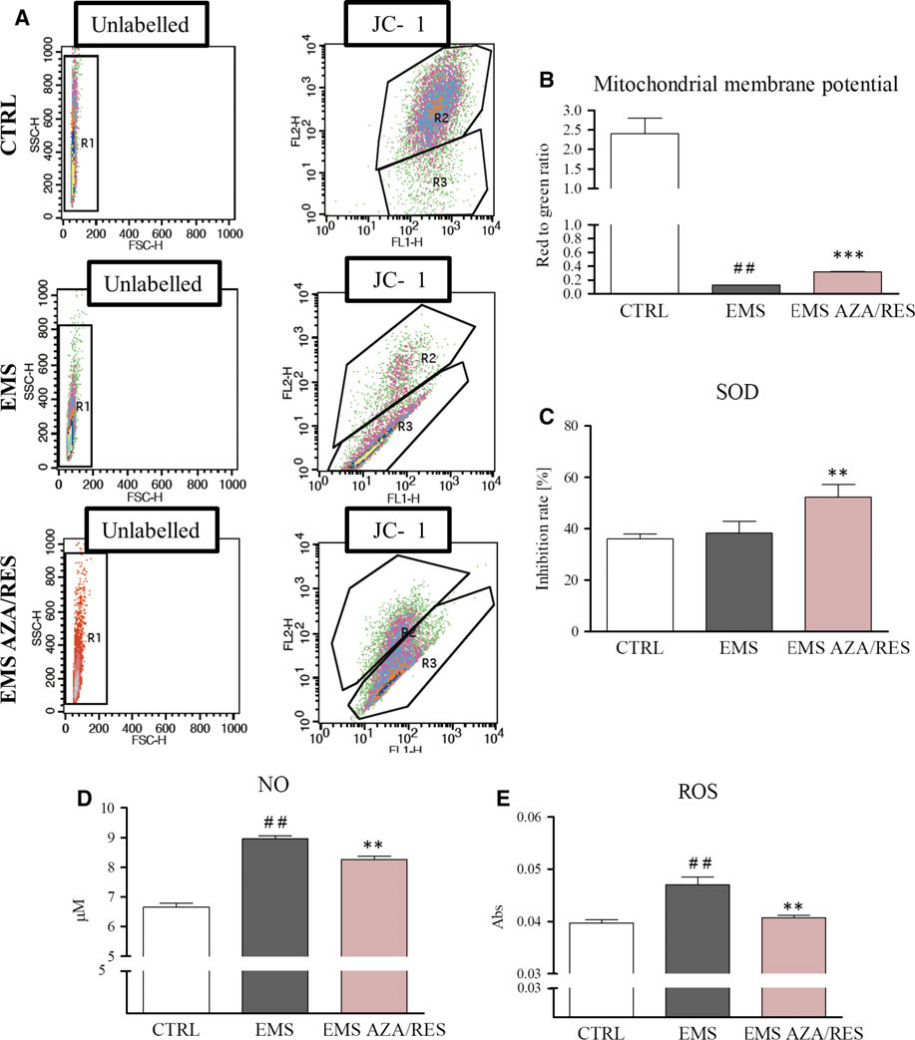
Evaluation of oxidative stress factors’ accumulation during osteogenic differentiation. Cells cultured in osteogenic medium for 1 day were subjected to flow cytometer analysis. Representative graphs of obtained data (A) and their quantification (B) revealed decreased MMP in EMS cells. Furthermore, activity of SOD (C) was assessed with spectrophotometric assay. NO (D) and ROS (E) analysis revealed that AZA/RES treatment ameliorates oxidative stress in treated cells. Results expressed as mean ± SD. Statistical significance indicated as asterisk (*) when comparing the result to ASC_EMS_, and as hashtag (#) when comparing to ASC_CTRL_. ##*P *<* *.01, **P <* *.01, ***P <* *.001

In order to evaluate mitochondrial membrane potential (MMP) with a flow cytometer, cells were subjected to analysis after day 1 of differentiation to avoid machine breakdown. Representative plots are shown in Figure 5A. MMP was significantly down‐regulated in ASC_EMS_ (Figure 5B, *P* < .01), although AZA/RES treatment resulted in increased MMP (*P *<* *.001). Antioxidative capacity of cells was estimated by the evaluation of SOD activity. No differences were noted between control and EMS groups, whereas ASC_EMS AZA/RES_ was characterized by an enhanced SOD activity (Figure 5C, *P* < .01). Accumulation of NO was increased in ASC_EMS_ (Figure 5D, *P* < .01), although AZA/RES treatment diminished its levels (*P *<* *.01). Analogous phenomenon was observed in the measurement of ROS levels as shown in Figure 5E.

### 3.6 Senescence and apoptosis

**Figure 6:**
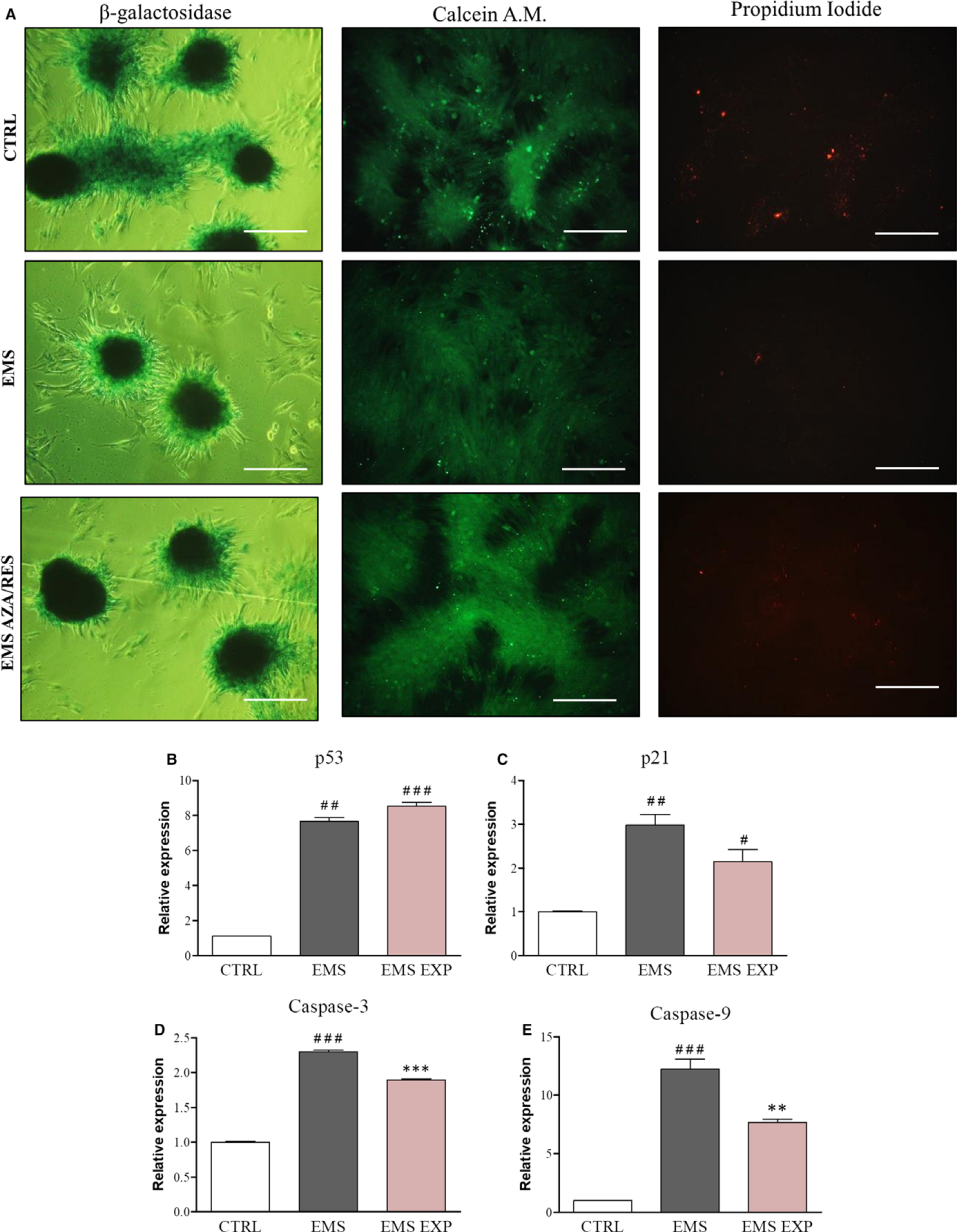
Investigation of apoptosis and senescence in cultures. Cultures were stained for β‐galactosidase accumulation, visualization of live (calcein A.M.) and apoptotic (propidium idodide) cells (A). Furthermore, using RT‐PCR the expression of apoptosis‐related genes including p53 (B), p21 (C), caspase‐3 (D) and caspase‐9 (E) was assessed. Results expressed as mean ± SD. Statistical significance indicated as asterisk (*) when comparing the result to ASC_EMS_, and as hashtag (#) when comparing to ASC_CTRL__._ ##*P *<* *.01, ###*P *<* *.001, **P <* *.01, ***P <* *.001. Magnification ×100, scale bar: 250 μm

For visualization of senescence in cultures, staining for β‐galactosidase was performed (Figure 6A). All formed bone nodules were characterized by dye accumulation. Furthermore, the greatest number of dead cells was noted in the control group as indicated in Figure 6A. ASC_EMS_ were characterized by diminished dye accumulation and number of dead cells which indicated early osteogenesis. Both ASC_CTRL_ and ASC_EMS AZA/RES_ displayed robust β‐galactosidase accumulation and number of dead cells as cells in those groups undergo apoptosis and formed a mineralized matrix. We further examined the expression of apoptotic‐related genes during osteogenesis. We found increased mRNA levels of p53 in ASC_EMS_ in comparison to ASC_CTRL_ (Figure 6B). No differences were noted between ASC_EMS_ and ASC_EMS AZA/RES._ Expression of p21 was up‐regulated in ASC from EMS horses (Figure 6C, *P* < .01); however, AZA/RES treatment reduced its levels in comparison to untreated cells (*P *<* *.05). Similar phenomenon was noted in the case of caspase‐3 (Figure 6D) and caspase‐9 (Figure 6E) expression.

### 3.7 Evaluation of ER stress

**Figure 7:**
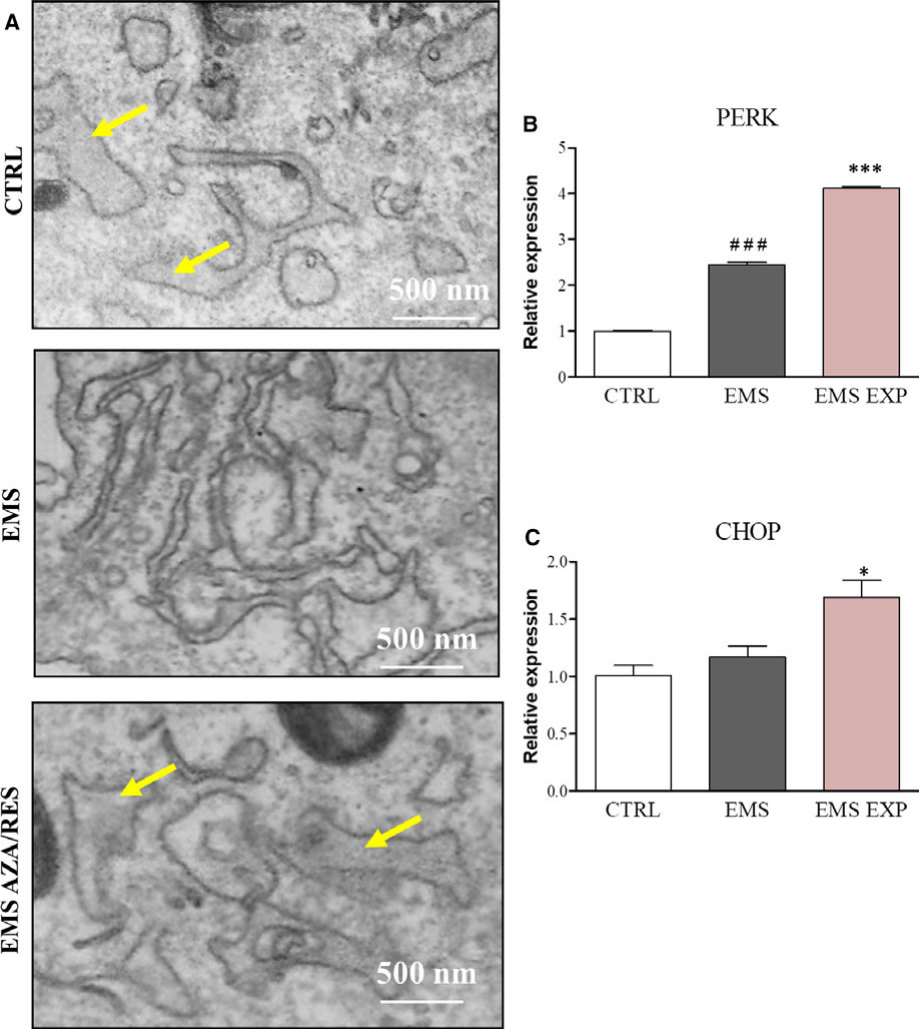
ER stress during osteogenic differentiation. Using TEM we investigated the ER ultrastructure (A) and expression of genes involved in ER stress pathway: PERK (B) and CHOP (C). Results expressed as mean ± SD. Statistical significance indicated as asterisk (*) when comparing the result to ASC_EMS_, and as hashtag (#) when comparing to ASC_CTRL__._ ###*P *<* *.001, *P <* *.05, ***P <* *.001

As it was shown that ER stress is involved in the progression of osteogenesis, we decided to investigate ER ultrastructure and expression of genes related to ER stress pathway. TEM images indicated that ER underwent severe ultrastructural changes in ASC_CTRL_ and ASC_EMS AZA/RES._ In those groups swelling of the ER lumen was observed. Expression of PERK was significantly increased in EMS group in comparison to healthy, control cells (Figure 7B, *P* < .001). Furthermore, PERK expression was markedly increased after AZA/RES treatment (*P *<* *.001) in comparison to untreated cells. No differences were noted in the expression of CHOP between ASC_CTRL_ and ASC_EMS_ (Figure 7C). However, its expression was up‐regulated in ASC_EMS AZA/RES_ in comparison to untreated cells (*P *<* *.05).

### 3.8 Evaluation of autophagy

**Figure 8:**
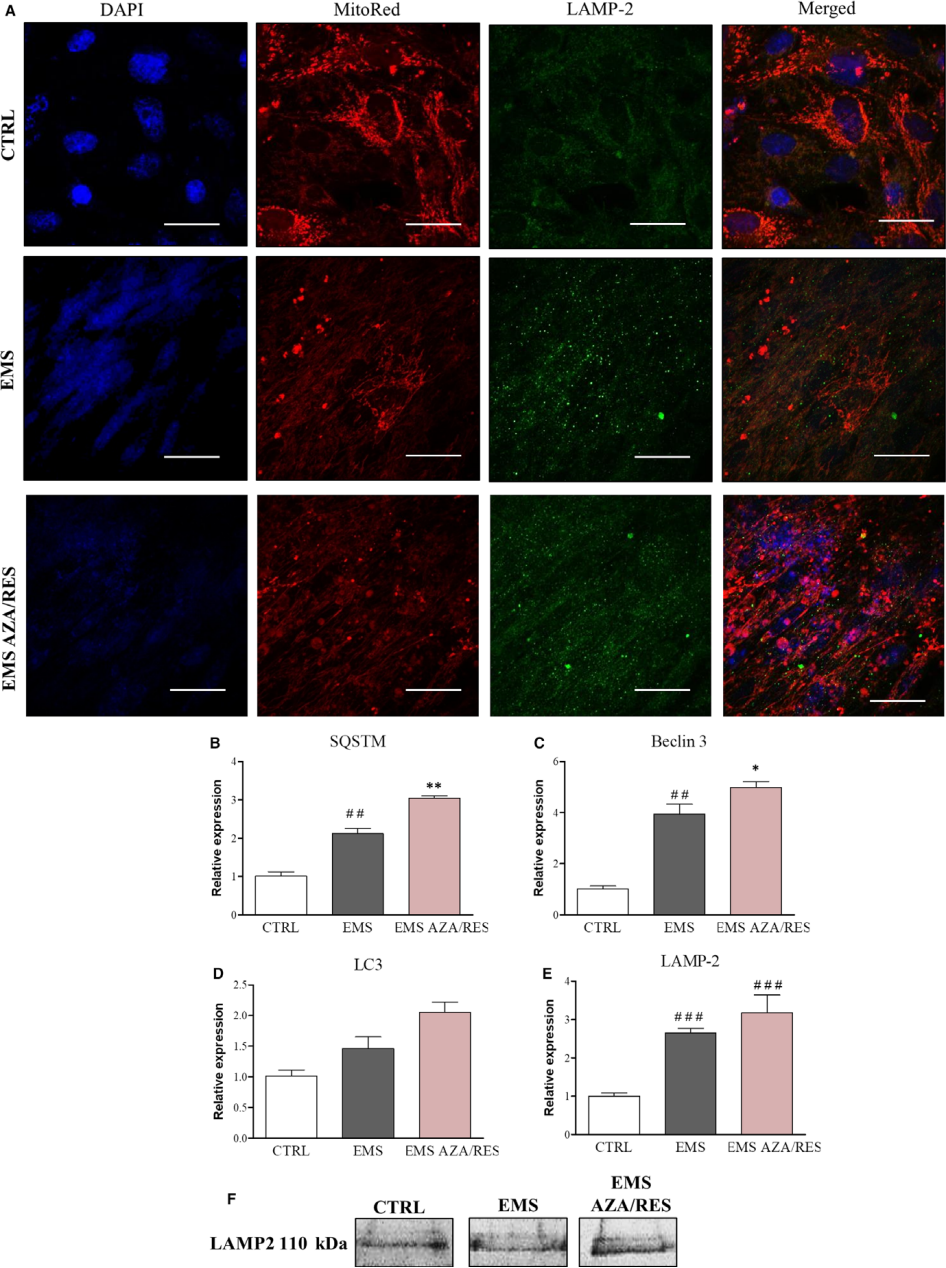
Evaluation of autophagy during osteogenic differentiation in control and AZA/RES conditions. Confocal photographs showing representative staining of mitochondria (MitoRed) and anti‐LAMP2 immunofluorescence (A). Using RT‐PCR the expression of SQSTM (B), Beclin 3 (C), LC3 (D) and LAMP‐2 (E). Furthermore, the amount of LAMP‐2 was visualized using western blot (F).Results expressed as mean ± SD. Statistical significance indicated as asterisk (*) when comparing the result to ASC_EMS_, and as hashtag (#) when comparing to ASC_CTRL_. ##*P *<* *.01, ###*P *<* *.001, *P <* *.05, **P <* *.01. Scale bar 40 μm

As autophagy plays an important role in cell survival and differentiation, we decided to investigate its levels during osteogenesis. For that reason, immunofluorescence for LAMP‐2 was performed (Figure 8A). The obtained results indicated that there was an increase in LAMP‐2 amount in ASC_EMS_ and in ASC_EMS AZA/RES._ Moreover, cells were also counterstained with MitoRed for MT visualization. The obtained results revealed increased MT activity in the control group which supports the data received with JC‐1 assay. SQSTM expression was increased in ASC_EMS_ (Figure 8B, *P* < .01) and AZA/RES treatment augmented its expression even more (*P *<* *.01). Similarly, the expression of Beclin 3 (Figure 8C) was also up‐regulated in those groups (*P *<* *.01 and *P *<* *.05, respectively). Interestingly, no differences were noted in the expression of LC3 (Figure 8D). LAMP‐2 expression was up‐regulated in ASC_EMS_ in comparison to ASC_CTRL_; however, no differences were found between ASC_EMS_ and ASC_EMS AZA/RES._ For that reason, we performed western blot for LAMP‐2 as well. Signal intensity was the greatest in ASC_EMS AZA/RES_ (Figure 8F)_._

### 3.9 Assessment of mitochondrial dynamics

**Figure 9:**
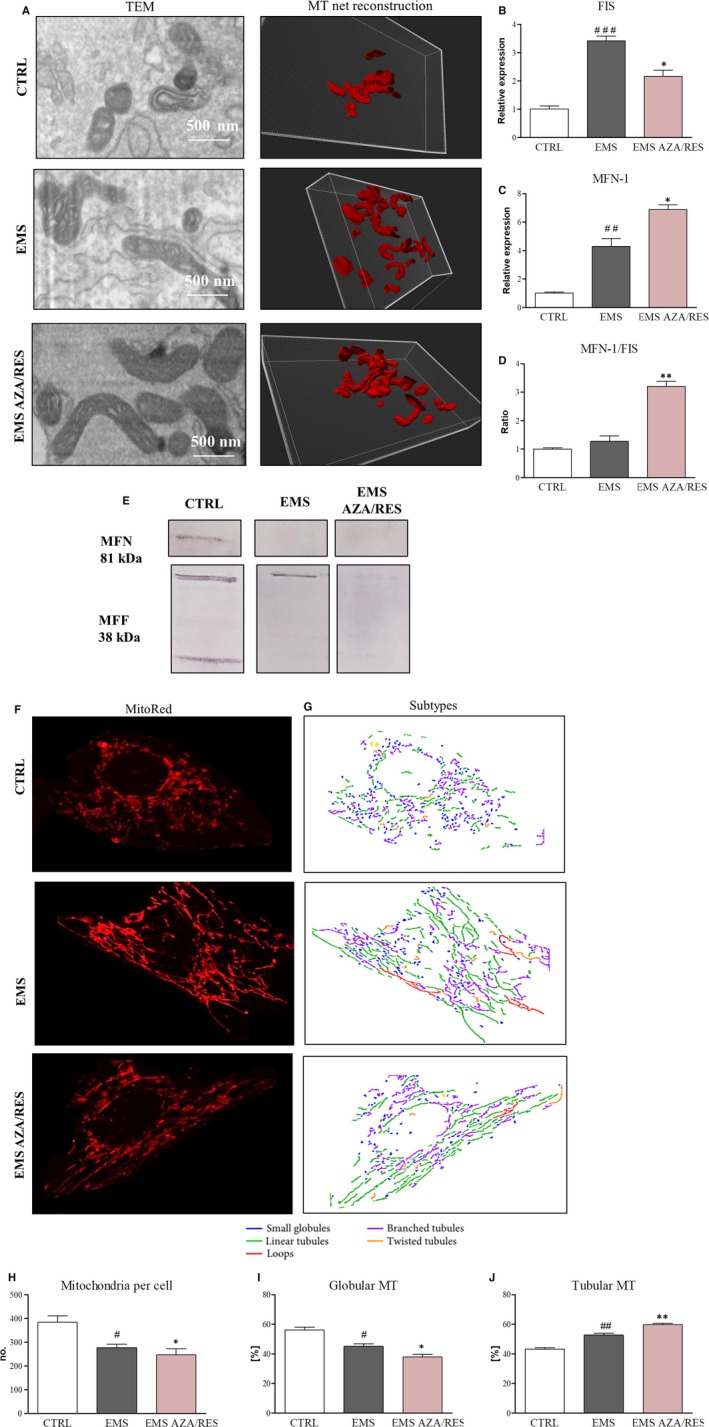
Evaluation of mitochondrial net morphology and dynamics. Using TEM pictures, reconstruction of mitochondrial net in Imaris Software was performed (A). Formation of elongated, connected mitochondria was noted in cells treated with AZA/RES. Furthermore, using RT‐PCR the expression of FIS (B) and MNF (C) was established. Moreover, the ratio of MNF‐FIS expression was calculated (D). Obtained results indicated that AZA/RES supports mitochondrial fusion over fission. MNF and MFF levels were further visualized using western blot (E). Pictures obtained from MitoRed staining (F) were subjected to microP (G) analysis. Obtained data were quantified and number of mitochondria in cell (H), amount of globular (I) and tubular (J) mitochondria were calculated. Results expressed as mean ± SD. Statistical significance indicated as asterisk (*) when comparing the result to ASC_EMS_, and as hashtag (#) when comparing to ASC_CTRL_. #*P *<* *.05, ##<.01, ###*P *<* *.001, *P <* *.05, **P <* *.01

Mitochondrial morphology was evaluated using TEM microscope and Imaris analysis (Figure 9A). TEM photographs indicated an increased fusion in ASC_EMS AZA/RES._ Those results were further supported by creating a 3D reconstruction of the mitochondrial net in Imaris software, based on FIB‐TEM images. RT‐PCR revealed increased expression of FIS in ASC_EMS_ (Figure 9B, *P* < .001) and decreased FIS mRNA amount after AZA/RES treatment (*P *<* *.5). Furthermore, expression of MFN was increased in ASC_EMS_ in comparison to control cells (Figure 9C, *P* < .01). The ratio of MFN/FIS expression was up‐regulated in AZA/RES group which indicates increased mitochondrial fusion (Figure 9D). Western blot for MFN indicated its increased synthesis in ASC_CTRL_ (Figure 9E). Furthermore, the shape of mitochondrial net was visualized by MitoRed (Figure 9F). Obtained photographs were subjected to microP software analysis (Figure 9G). In accordance with the data generated by the software, ASC_EMS_ and ASC_EMS AZA/RES_ were characterized by decreased MT number per cell (Figure 9H). Moreover, the number of globular MT was diminished in ASC_EMS_ in comparison to ASC_CTRL_ (Figure 9I, *P* < .05). Treatment of ASC_EMS_ with AZA/RES decreased globular organelles’ number (*P *<* *.05). An opposite trend was observed in the amount of tubular MT (Figure 9J). Their number was increased in ASC_EMS_ in comparison to ASC_CTRL_ (*P *<* *.05). However, ASC_EMS_ preconditioned with AZA/RES were characterized by increased tubular organelles in comparison to untreated cells (*P *<* *.05).

### 3.10 Evaluation of mitophagy

**Figure 10:**
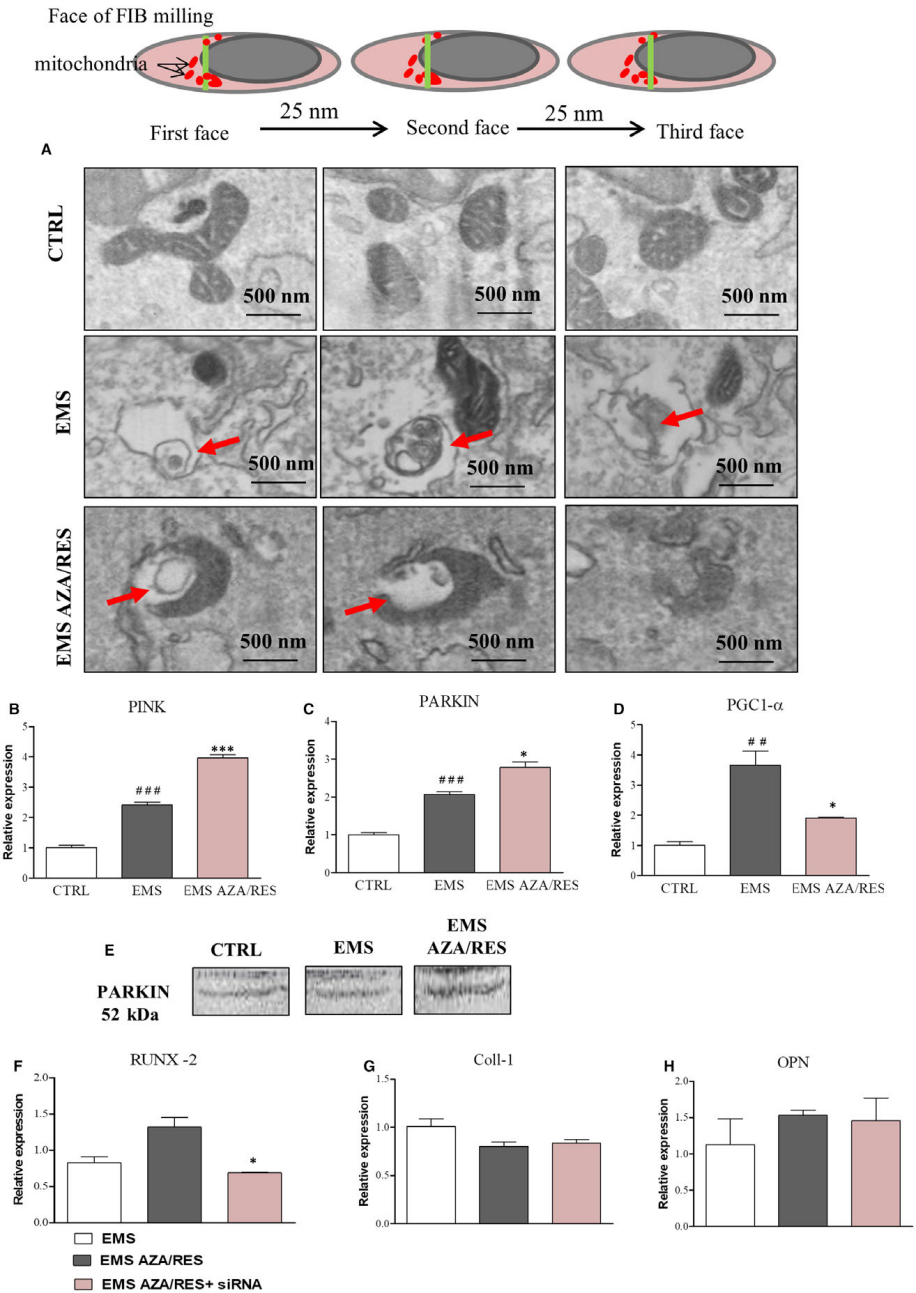
Evaluation of mitophagy during osteogenic differentiation in control and AZA/RES treated cells. Using TEM‐FIB, cells were sliced every 25 nm to visualize the formation of mitophagosomes (A, indicated with red arrows). Using RT‐PCR the expression of mitophagy related genes: PINK (B) and PARKIN (C) was investigated. Moreover, we assessed the expression of PGC1‐α as a marker for mitochondrial biogenesis (D). Amount of protein PARKIN was visualized with western blot (E). To confirm that increased mitophagy is responsible for the beneficial effects of AZA/RES, using siRNA expression of PARKIN was silenced, and expression of RUNX‐2 (F), Coll‐1 (G) and  OPN (H) was investigated. Results expressed as mean ± SD. Statistical significance indicated as asterisk (*) when comparing the result to ASC_EMS_, and as hashtag (#) when comparing to ASC_CTRL_. ##*P *<* *.01, ###*P *<* *.001, *P <* *.05, ***P <* *.001

Mitophagy was assessed using FIB‐TEM. Increased formation of mitophagosomes was noted in ASC_EMS_ and ASC_EMS AZA/RES_ (Figure 10A). To support the qualitative results from the images, we performed RT‐PCR for PINK and PARKIN. Indeed, increased expression of PINK was noted in ASC_EMS_ in comparison to ASC_CTRL_ (Figure 10B, *P* < .001). AZA/RES significantly induced PINK expression in treated cells (*P *<* *.001). Similarly, PARKIN mRNA amount was augmented in ASC_EMS_ in comparison to ASC_CTRL_ (Figure 10C, *P* < .001) and AZA/RES significantly induced PARKIN expression in the experimental group (Figure 10C, *P *<* *.05). PGC1a was up‐regulated in ASC_EMS_ (Figure 10D, *P* < .01); however, AZA/RES diminished its expression (*P *<* *.05). Western blot for PARKIN supported RT‐PCR data as it indicated an increased PARKIN amount in the experimental group. To confirm, if mitophagy is responsible for enhanced osteogenesis in AZA/RES group, we performed PARK silencing and analysed genes expression with RT‐PCR. After silencing PARKIN in ASC_EMS AZA/RES_, significantly decreased RUNX 2 expression was observed (Figure 10F, *P* < .05). Interestingly, no differences were observed in collagen II (Figure 10G) and OPN (Figure 10H) expression levels which indicate that AZA/RES improves osteogenesis by the regulation of RUNX 2 activity.

### 3.11 Immunomodulatory properties of osteoblastic cells

**Figure 11:**
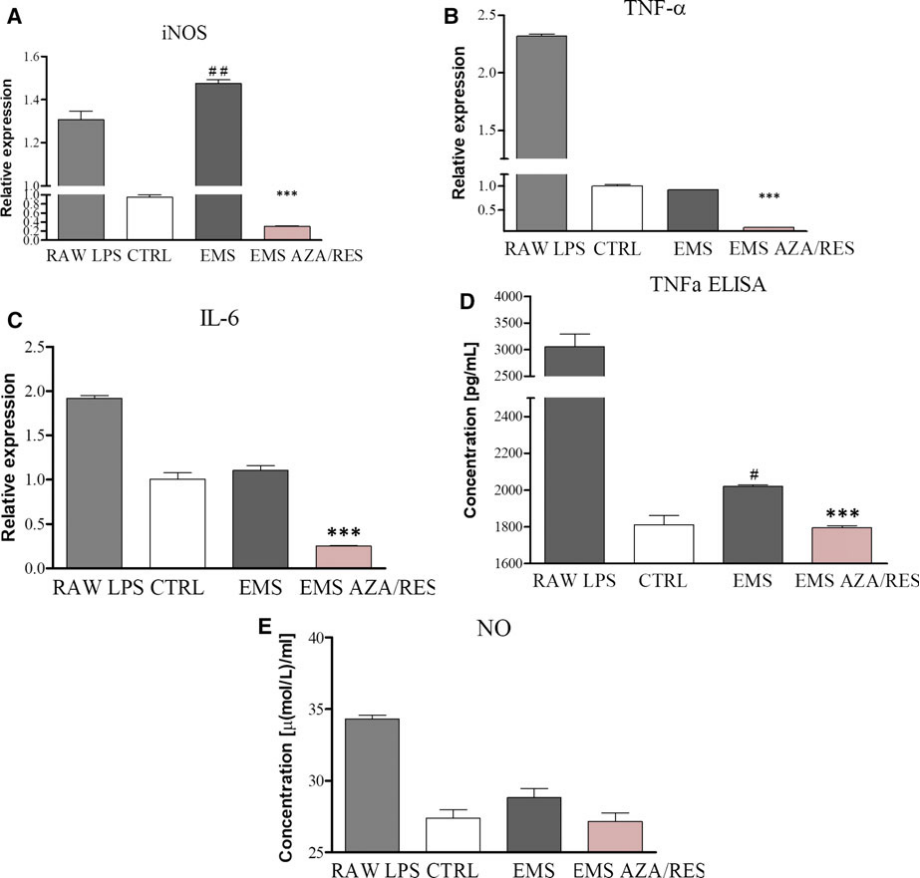
The effect of AZA/RES on the activation status of RAW 264.7 macrophages. In order to perform the experiments, ASC after day 5 of osteogenesis were co‐cultured with LPS‐treated macrophages. The effect of AZA/RES pre‐treatment on iNOS (A), TNF‐α (B) and IL‐6 (C) gene expression was evaluated by the means of RT‐PCR. Furthermore, supernatants were collected and subjected for TNF‐α (D) and NO (E) analysis. Results expressed as mean ± SD. Statistical significance indicated as asterisk (*) when comparing the result to ASC_EMS_, and as hashtag (#) when comparing to ASC_CTRL_. #*P *<* *.05, ##<.01, ***P <* *.001

In order to evaluate the ability of cells to modulate immune response, ASC after day 5 of osteogenesis (osteoblastic cells) were co‐cultured with RAW 264.7 macrophages. Using RT‐PCR we evaluated the expression levels of iNOS, TNF‐α and IL‐6 in RAW 264.7 cell line. iNOS expression in ASC_EMS_ group exceed the levels observed not only in ASC_CTRL_ but also in LPS‐treated macrophages (Figure 11A). However, treatment of ASC_EMS_ with AZA/RES significantly reduced iNOS expression in stimulated macrophages (*P *<* *.001). TNF‐α mRNA levels were comparable between control and EMS groups (Figure 11B). Preconditioning of cells with AZA/RES resulted in significantly decreased expression of TNF‐α in the experimental group when compared to ASC_EMS_ (*P *<* *.001). A similar trend was noted in the expression of IL‐6 as indicated in Figure 11C. Furthermore, TNF‐α secretion was analysed with ELISA assay (Figure 11D). Increased amount of TNF‐α was noted in ASC_EMS_ in comparison to the control group. However, AZA/RES treatment significantly down‐regulated the TNF‐α level (*P *<* *.001). Using spectrophotometric assay, the amount of NO was investigated; however, no statistically significant differences were noted in the investigated groups (Figure 11E).

### 3.12 Epigenetic changes during differentiation

**Figure 12:**
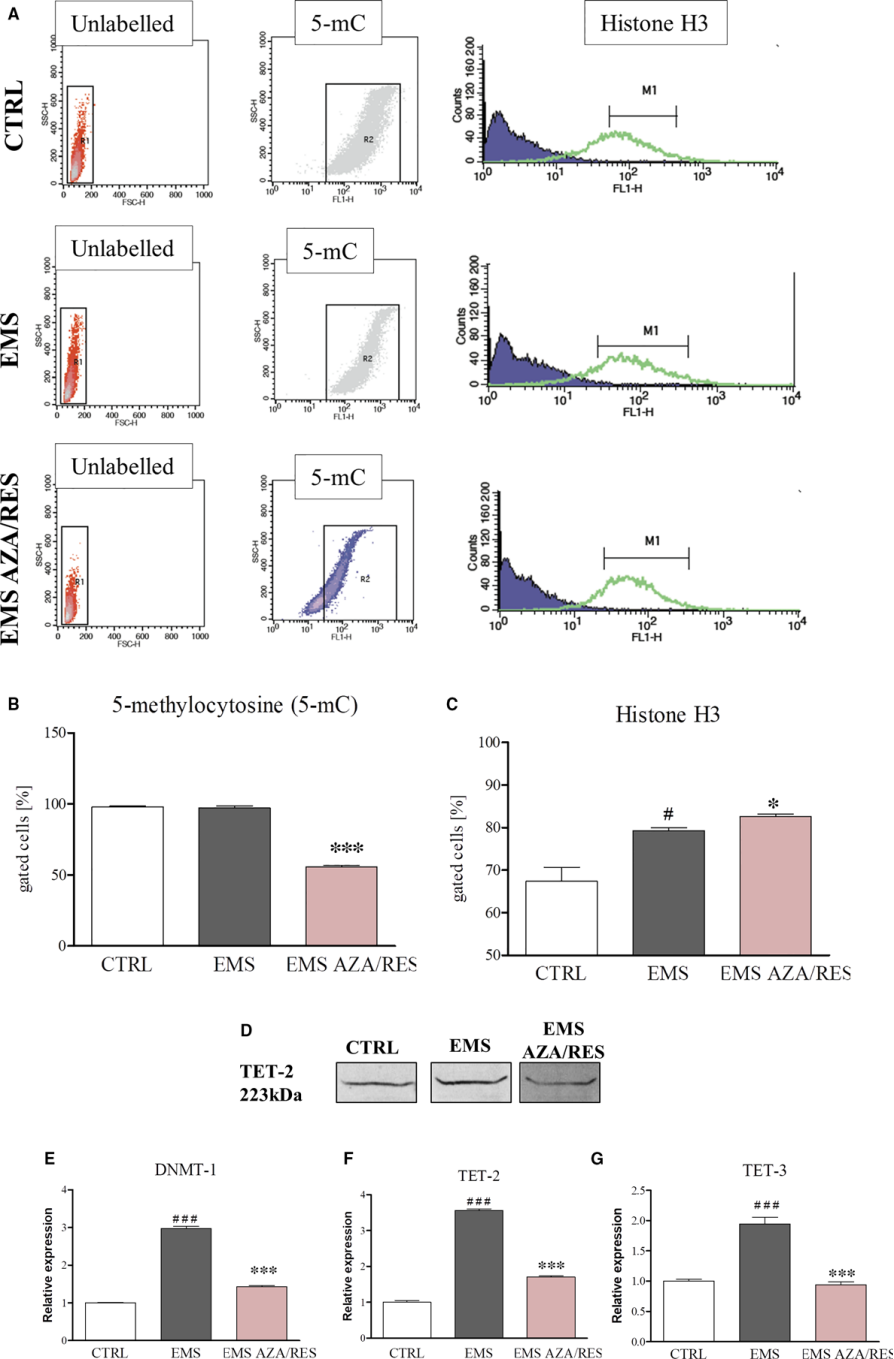
Evaluation of epigenetic alternations after AZA/RES treatment. Representative graphs are showing flow cytometry results (A). 5‐mC (B)‐positive cell number was significantly decreased after AZA/RES treatment. On the other hand, AZA/RES augmented the number of histone H3‐positive cells (C). TET‐2 protein levels were visualized using western blot (D). Moreover, the expression of DNMT‐1 (E), TET‐2 (F) and TET‐3 (G) was tested by RT‐PCR analysis. Results expressed as mean ± SD. Statistical significance indicated as asterisk (*) when comparing the result to ASC_EMS_, and as hashtag (#) when comparing to ASC_CTRL_. #*P *<* *.05, ###*P *<* *.001, *P <* *.05, ***P <* *.001

In order to investigate epigenetic alternations in cells, we analysed with flow cytometer 5‐mC and histone H3 accumulation. No difference in 5‐mC‐positive cells was noted between ASC_CTRL_ and ASC_EMS;_ however, after treatment with AZA/RES 5‐mC level was significantly diminished (Figure 12B, *P* < .001). H3‐positive cell number was increased in ASC_EMS_ in comparison to the control group and AZA/RES treatment enhanced H3‐positive cells even more (Figure 11C, *P* < .05). Using western blot, the amount of TET‐2 was investigated. In accordance with the obtained results, cells treated with AZA/RES were characterized by the lowest TET‐2 synthesis (Figure 11D). Expression of DNMT1 was assessed using RT‐PCR and obtained data revealed its increased level in ASC_EMS_ in comparison with control cells (Figure 11E, *P* < .001). However, treatment of ASC_EMS_ with AZA/RES resulted in significantly decreased DNMT1 expression (*P *<* *.001). Analogues phenomenon was observed in the expression level of TET‐2 (Figure 11F) and TET‐3 (Figure 11G) as AZA/RES treatment significantly decreased their mRNA amount (*P *<* *.001).

## 4 DISCUSSION

The present study has demonstrated that *ex vivo* treatment of ASC_EMS_ with a combination of AZA/RES improved osteogenic differentiation of these cells in comparison to non‐treated cells. Enhanced osteogenesis was accompanied by the higher expression of Runx2, BMP‐2, OPN, Col‐1 and miR‐451. Greater calcification, ie higher levels of calcium and phosphorus and increased number of osteo‐nodules, was positively correlated with their larger size. Interestingly, ALP activity was significantly decreased by AZA/RES, which indicated their silencing effect on early osteogenesis markers. Moreover, co‐culture of AZA/RES‐treated ASC_EMS_ with RAW 264 macrophages improved their immunomodulatory effect, as reduced expression of iNOS, TNF‐α and Il‐6 was observed. The results indicated that the *ex vivo* application of AZA/RES reduced OS, senescence and improved mitochondrial dynamics, thereby improving osteogenic differentiation potential of physiologically deteriorated ASC_EMS_. Moreover, increased mitochondrial dynamics, which correlated with higher osteogenic potential, was positively associated with the activation of Parkin‐dependent mitophagy.

Endocrine disorders, including MetS in humans or EMS, have been shown to deteriorate multipotency and multilineage differentiation potential of ASCs. It was demonstrated that excessive accumulation of OS factors, ie ROS and NO, together with significantly decreased antioxidant protection, might be associated with reduced osteogenic differentiation potential and extracellular matrix formation.^28^, ^33^ In turn, many studies including ours, have shown that RES significantly attenuates OS by modulating SOD activity and mitochondrial dynamics.^34^, ^35^, ^36^ Consistent with these studies, we observed that the *ex vivo* application of AZA/RES significantly reduced the level of both ROS and NO. Simultaneously, we recorded enhanced SOD activity and increased MMP. Furthermore, it was shown that the *ex vivo* application of AZA/RES improved the viability of ASC_EMS_, as we observed decreased amounts of p21, Cas‐3 and Cas‐9 mRNA transcripts. Our results are consistent with the study of Kao et al,^37^ who showed that RES treatment promoted osteogenic differentiation potential in murine pluripotent stem cells. In addition, Shakibaei et al^38^ discovered that RES improved osteogenic differentiation through Sirt‐1/Runx2. Moreover, it has been found that RES inhibits Akt signalling by increasing PP1α activity and enhancing 2‐DG‐induced ER stress signalling, which in turn is required for the differentiation of several tissues, including osteoblasts.^39^ In this study, we observed a greater expression of CHOP and PERK in ASC_EMS_ in the presence of AZA/RES, which corresponded to ultrastructural ER abnormalities. Moreover, a positive correlation of CHOP/PERK and BMP‐2 expression was found. This is in line with the results of Zhang et al,^40^ who showed that ER stress inducible factor cysteine‐rich with EGF‐like domains 2 (Creld2) was involved in mediating BMP‐dependent osteogenesis in mesenchymal stem cells.

It has been shown that OS combined with epigenetic genome modifications is a crucial factor inducing ageing and senescence in MSCs,^25^, ^29^, ^33^ leading to permanent growth arrest and final induction of apoptosis. Recent study has suggested that allogeneic stem cells may elicit an immune response in a recipient animal and that allogeneic donor MSCs are not fully immune‐privileged, as previously claimed.^41^ Therefore, we have verified the hypothesis whether *ex vivo* treatment of ASC_EMS_ with AZA/RES may reverse their aged phenotype and improve osteogenic differentiation. The current study demonstrated that the combination of AZA/RES reduced 5‐methylocysteine (5‐mC) level, increased histone deacetylase 3 (H3) expression and reduced DNA methyltransferase‐1 (DNMT1) expression. A reduced activity of DNMT1, which is responsible for DNA methylation during replication in the course of osteogenic differentiation, has been previously reported.^42^ Furthermore, excessive DNMT activity was linked to apoptosis and DNMT inhibition; however, it exerted neuroprotective effects on neuronal cells.^43^, ^44^ Moreover, Carpio et al^45^ discovered that H3 supported endochondral bone formation by controlling cytokine signalling and matrix remodelling, which was consistent with our results. Moreover, it was shown that DNA‐demethylation was associated with the induction of the TET gene expression, which initiated the conversion of nuclear 5‐methylcyt‐osine (5 mC) to 5‐hydroxymethylcytosine (5 hmC). In our previous work, we showed that 5‐AZA reversed the aged phenotype of human ASCs isolated from elderly patient by increasing the proliferation, viability and metabolic activity through a higher BCL‐2/BAX ratio combined with reduced expression of p21/p53/Cas‐9.^25^ However, the expression of TET2 and TET3 was comparable between the control and AZA/RES groups in our study. Recent studies suggested that the expression of TET 2 and TET3 might be impaired under pathological conditions, ie haematological malignancies, melanoma and/or neurodegeneration.^46^, ^47^ However, it should be taken into account that our data concerns the fifth day of osteogenesis, and TET proteins may be switched on earlier in the AZA/RES group because epigenetic alternations are necessary for the differentiation process.

Oxidative stress is recognized as a major factor contributing to the deterioration of ASC multipotency, isolated from patients with several conditions, including obesity, diabetes and ageing.^20^, ^21^ Our previous research has shown that autophagy in ASC_EMS_ serves as a protective mechanism that allows to maintain cellular homeostasis and stemness. We have confirmed increased mitophagy in ASC_EMS_ treated with AZA/RES using RT‐PCR, TEM‐FIB and confocal microscopy. In this study, we observed the overexpression of SQSTM1 (p62), Beclin 3, LAMP‐2 and LC3 in ASC_EMS_. Our results indicate enhanced lysosomal activity in ASC_EMS_ treated with AZA/RES because the up‐regulation of LAMP‐2 and LC3 occurs during last stages of autophagy. These data were confirmed by immunofluorescence, as abundant accumulation of cytoplasmic LAMP‐2‐positive granules was noted in ASCs_EMS_. These results are consistent with several studies indicating the role of RES in autophagy modulation.^22^, ^48^, ^49^ In addition to its antioxidant properties, RES promotes autophagic turnover under stress as well as differentiation of physiologically impaired ASCs. Moreover, the balance between mitochondrial fusion and fission is required to maintain energy balance in cells. Several studies have demonstrated that the expression of MNF2 decreased in the kidney, myocardium and retina in diabetes patients, which was associated with excessive apoptosis regulated by BAX/BCL‐2 expression.^42^, ^50^, ^51^ Recently, Robb et al^53^ have shown that RES stimulates mitochondrial fusion and cell growth through mitofusin activity. Similar to the latter study, we observed an up‐regulation of MNF with a simultaneously decreased FIS expression. These findings were supported by 3D projection of MT based on TEM photographs. A tendency to form the so‐called tubular morphoptype in ASCs_EMS_ treated with AZA/RES was noted.

Mitophagy is a process of mitochondrial quality control regulated by mitochondrial kinase (PINK1) and ubiquitin ligase (PARKIN). In this pathway, PINK1 senses mitochondrial damage and activates Parkin by phosphorylation and ubiquitination. Impaired MT, characteristic of ASCs_EMS_, are eliminated in PINK/PARKIN‐dependent manner, as previously shown by our group.^27^, ^28^ The research conducted by Kornicka et al^34^ showed that RES incorporated into polyurethane‐polylactide‐based material induced PINK/PARKIN expression in humans ASCs, which was associated with increased expression of osteogenic markers. We observed in the current study that the exposure of ASC_EMS_ to AZA/RES resulted in an increased expression of PINK and PARKIN. The TEM‐FIB technique allowed us to visualize and analyse single MT ultrastructure and formation of autophagic vacuoles. Mitophagy in ASC_EMS_ may be essential for cell survival during osteogenic differentiation. This hypothesis may be supported by PARKIN knockdown, which resulted in the down‐regulation of RUNX and Coll‐1; this in turn underlines its role in the course of osteogenic differentiation. Our previous research demonstrated that ASC_EMS_ had impaired mitophagy, as decreased PGC1α, PARKIN and PDK4 activities together with reduced osteogenic differentiation potential were recorded.^28^ Thus, the obtained data indicate that pre‐treatment of ASC_EMS_ with AZA/RES enhances osteogenic properties of these cells via increased mitophagy.

The present study demonstrates that the *ex vivo* treatment of ASC_EMS_ with AZA/RES improves viability, metabolic activity and mitochondrial potential by reducing OS, thereby improving osteogenic differentiation potential. Clinical application of ASC_EMS_ in orthopaedic therapies is seriously limited and flawed because we have previously provided evidence that these cells are characterized by reduced multilineage differentiation potential. Therefore, we speculate that the *ex vivo* treatment of ASC_EMS_ with AZA/RES may become a necessary intervention in order to increase the osteogenic capacity of these cells, as it results in a higher expression of Runx2, BMP‐2, OPN, Col‐1 and miR‐451. Moreover, ASCs_EMS_ treated with AZA/RES were characterized by enhanced immunomodulatory properties. Decreased levels of iNOS, TNF‐α and Il‐6 were observed in co‐culture with RAW 264.7.

In summary, the osteogenic potential of ASC_EMS_ can be improved by *ex vivo* application of AZA/RES. Further studies confirming the clinical functionality of AZA/RES‐treated ASC_EMS_ are required to develop effective cell therapy.

## CONFLICT OF INTEREST

The authors declare that there is no conflict of interest.
